# Structural divergence of plant TCTPs

**DOI:** 10.3389/fpls.2014.00361

**Published:** 2014-07-29

**Authors:** Diego F. Gutiérrez-Galeano, Roberto Toscano-Morales, Berenice Calderón-Pérez, Beatriz Xoconostle-Cázares, Roberto Ruiz-Medrano

**Affiliations:** Department of Biotechnology and Bioengineering, CINVESTAV-IPNMexico DF, Mexico

**Keywords:** Translationally Controlled Tumor Protein, *Arabidopsis thaliana*, evolution, phylogeny, GTP-binding pocket

## Abstract

The Translationally Controlled Tumor Protein (TCTP) is a highly conserved protein at the level of sequence, considered to play an essential role in the regulation of growth and development in eukaryotes. However, this function has been inferred from studies in a few model systems, such as mice and mammalian cell lines, Drosophila and Arabidopsis. Thus, the knowledge regarding this protein is far from complete. In the present study bioinformatic analysis showed the presence of one or more *TCTP* genes per genome in plants with highly conserved signatures and subtle variations at the level of primary structure but with more noticeable differences at the level of predicted three-dimensional structures. These structures show differences in the “pocket” region close to the center of the protein and in its flexible loop domain. In fact, all predictive TCTP structures can be divided into two groups: (1) AtTCTP1-like and (2) CmTCTP-like, based on the predicted structures of an Arabidopsis TCTP and a *Cucurbita maxima* TCTP; according to this classification we propose that their probable function in plants may be inferred in principle. Thus, different *TCTP* genes in a single organism may have different functions; additionally, in those species harboring a single *TCTP* gene this could carry multiple functions. On the other hand, *in silico* analysis of *AtTCTP1*-like and *CmTCTP*-like promoters suggest that these share common motifs but with different abundance, which may underscore differences in their gene expression patterns. Finally, the absence of *TCTP* genes in most chlorophytes with the exception of *Coccomyxa subellipsoidea*, indicates that other proteins perform the roles played by TCTP or the pathways regulated by TCTP occur through alternative routes. These findings provide insight into the evolution of this gene family in plants.

## Introduction

The Translationally Controlled Tumor Proteins, or TCTP, constitute a protein family found exclusively in eukaryotes, which are central for growth regulation (Bommer et al., 2002). Sequence comparison of TCTP sequences reveals a high degree of conservation among all eukaryotic phyla (Hinojosa-Moya et al., 2008). Such conservation, as well as its nearly ubiquitous expression underscores its essential role in the regulation of growth and development of different organisms (Thaw et al., 2001; Bommer and Thiele, 2004; Amson et al., 2013).

Currently, work on TCTP has focused mostly on animals, particularly models like human, mice and Drosophila, which has shown it to be involved in several biological processes such as cell growth, cell cycle progression, differentiation, malignant transformation, protection against various stress conditions and apoptosis (Amson et al., 2013). Studies on TCTP function in plants have been conducted with few experimental systems, mostly Arabidopsis. Although the molecular function of TCTP in plants has not been completely established yet, it is likely that in broad terms its function is similar, e.g., growth and developmental regulation since such function appears to be conserved across kingdoms; indeed a Drosophila *TCTP* mutant can be rescued with the corresponding Arabidopsis TCTP gene, and vice versa (Brioudes et al., 2010). Extant data suggest that *TCTP* is constitutively expressed at high levels in most tissues in different plants species; there is also evidence that TCTP expression is affected by a variety of conditions (Bommer and Thiele, 2004; Nagano-Ito and Ichikawa, 2012; Amson et al., 2013). Indeed, *TCTP* mRNA levels vary considerably in response to a wide range of extracellular stimuli and in multiple seemingly unrelated cellular processes (Bommer and Thiele, 2004).

The first plant *TCTP* mRNA sequence was obtained in *Medicago sativa* (Pay et al., 1992). The notion that its expression in plants correlates positively with growth was supported by the fact that *TCTP* mRNA accumulated in the root cap of *Pisum sativum*, a region undergoing continuous cell division (Woo and Hawes, 1997). Furthermore, possible functions related to photoperiodism and flowering in *Pharbitis nil* have been suggested (Sage-Ono et al., 1998). *TCTP* is regulated in response to a wide range of stimuli, such as aluminum (Ermolayev et al., 2003), damage caused by Hg^2+^ (Wang et al., 2012) and NaCl (Vincent et al., 2007; Cao et al., 2010), heat, cold, and drought (Kim et al., 2012; Li et al., 2013), as well as growth regulators such as auxins, ABA (Berkowitz et al., 2008; Cao et al., 2010; Kim et al., 2012), and methyl jasmonate (Li et al., 2013). TCTP also seems to be involved in response to pathogens such as *Pseudomonas syringae* (Jones et al., 2006) and *Erysiphe grαminis*, the causal agent of powdery mildew in wheat (Li et al., 2010).

On the other hand, TCTP has been detected in the phloem sap of *Ricinus communis* and *Cucurbita maxima*, suggesting its phloem long-distance movement. This in turn suggests that these proteins, at least in some species, act in a non-cell-autonomous manner (Barnes et al., 2004; Aoki et al., 2005; Hinojosa-Moya et al., 2006). It is worth mentioning that lupin *TCTP* mRNA is present in the phloem sap transcriptome, and the pumpkin *TCTP* mRNA has been found in phloem sap exudates (Rodriguez-Medina et al., 2011; Hinojosa-Moya et al., 2013).

High expression levels of this gene have also been detected in embryo and endosperm during embryo development in *Jatropha curcas* and *Ricinus communis*, indicating that *TCTP* participates in this process (Lu et al., 2007; Qin et al., 2011). Indeed, loss of function of Arabidopsis *AtTCTP1* leads to delayed embryo development (Brioudes et al., 2010). *AtTCTP1* is expressed throughout plant tissues and developmental stages, particularly in meristematic and expanding cells; moreover, and by analogy to Drosophila TCTP, it might act as a mediator of TOR activity via interaction with Rho GTPases in a similar manner to non-plant systems (Berkowitz et al., 2008), although the involvement of TCTP in the TOR pathway is still debated (Wang et al., 2008). Other results suggest that *AtTCTP1* controls mitotic growth and cell division but not cell expansion in plants (Brioudes et al., 2010) and cell cycle progression (Nakkaew et al., 2010).

**Figure 1:**
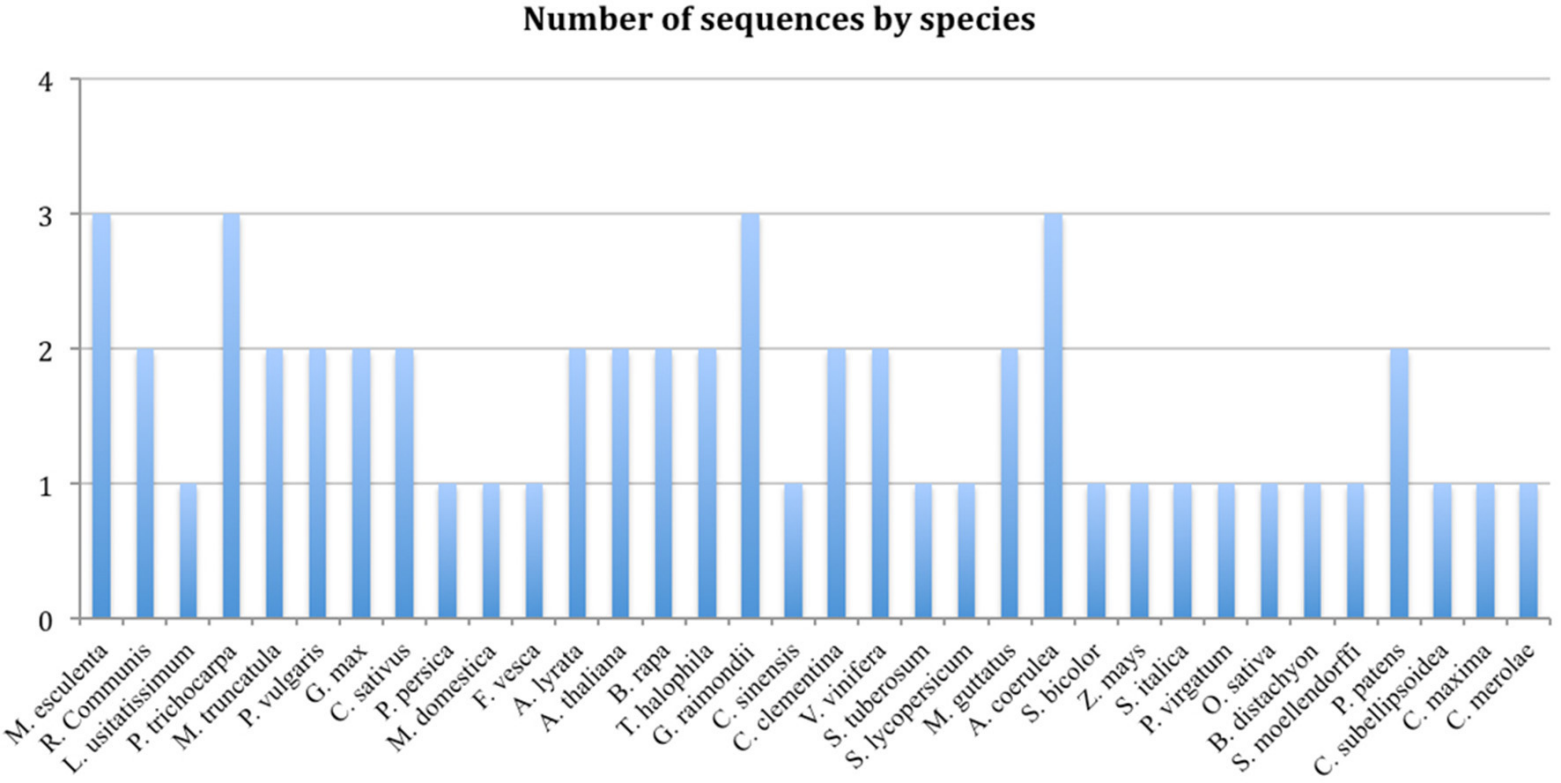
**Number of TCTP sequences by plant species (Source: Phytozome)**.

There are variable numbers of TCTP genes in different organisms, while mammals harbor the highest number of TCTP-like sequence per genome (for example, human, mouse and rat each harbor 3 *TCTP* genes and a larger number of pseudogenes), plants and fungi in general contain one or two *TCTP* genes (Hinojosa-Moya et al., 2008; Figure 1). It is possible that in organisms with multiple TCTP genes these are redundant; however, in some cases it is also possible that each performs non-overlapping or partially overlapping functions.

Currently, several *TCTP* genes have been isolated and cloned from different plants species. However, there are few studies regarding their functional characterization. The following table presents information about TCTP in different plant species and its identified function (Table 1). These published data suggest that TCTP not only regulates general growth but also carries out functions that are specific for plants, such as auxin homeostasis, regulation of stomatal closure, defense response to bacteria, positive regulation of microtubule depolymerization, drought recovery, pollen tube growth, among others. Based on this wide range of functions and the different TCTP versions present in plants, we suggest a specialization of functions (or division of labor) of this multifunctional protein. In the present work we propose that, based on an Arabidopsis (*AtTCTP1*) and a *Cucurbita maxima* (pumpkin) TCTP (*CmTCTP*) genes, their probable functions and predicted three-dimensional structures, there are two groups of TCTP genes, an AtTCTP1-like clade and a CmTCTP-like clade, and their respective functions may be inferred from such grouping. We also found that only one chlorophyte, among those whose genomes have been sequenced, harboring a TCTP gene, with possible implications on land plant evolution.

**Table 1:** **List of *TCTP* genes present in different plant species and their functions**.

**Species**	**Gene**	**Function**
	**Gene Bank accession number**	
*Jatropha curcas* (Physic Nut)	JcTCTP EF091818	Regulation of the endosperm development (Qin et al., 2011)
*Cucurbita maxima* (Pumpkin)	CmTCTP DQ304537	Non-cell-autonomous function and long-distance movement of phloem proteins (Aoki et al., 2005; Hinojosa-Moya et al., 2006).
*Arabidopsis thaliana* (Thale cress)	AtTCTP NM112537	Regulator of mitotic growth and cell cycle duration (Brioudes et al., 2010) Growth regulator, auxin homeostasis, root hair development and lateral root formation (Berkowitz et al., 2008) Drought tolerance, ABA-mediated stomatal closure, tubulin-, and calcium-binding properties (Kim et al., 2012)
*Fragaria ananassa* (Strawberry)	FaTCTP Z86091	Fruit ripening? (Lopez and Franco, 2006)
*Elaeis guineensis* (Oil palm)	EgTCTP AAQ87663.1	Cell growth and cell cycle progression (Nakkaew et al., 2010)
*Brassica oleracea* (Cabbage)	BoTCTP AF418663	Growth regulation and defense response to cold, high temperature, and salinity stresses (Cao et al., 2010).
*Pharbitis nil* (Morning glory)	PnTCTP AB007759	Photoperiodism, flowering? (Sage-Ono et al., 1998)
*Oriza sativa* (Rice)	OsTCTP BAA02151	Response to Hg^2+^ stress (Wang et al., 2012)
*Nicotiana tabacum* (Tobacco)	NtTCTP AF107842	Cell cycle progression (Brioudes et al., 2010)
*Triticum aestivum* (Wheat)	TaTCTP AF508970	Powdery mildew resistance? (Li et al., 2010)
*Ricinus communis* (Castor bean)	RcTCTP RCOM_1433410	Endosperm formation? (Lu et al., 2007)
*Vitis vinifera* (Grape Vine)	VvTCTP NC_012020	Response to water deficit and salinity (Vincent et al., 2007)
*Manihot sculenta* (Cassava)	MsTCTP AAM55492	Storage root formation (de Souza et al., 2004)
*Hevea brasiliensis* (Rubber tree)	HbTCTP JN200814	Response to ethrel, wounding, methyl jasmonate, low temperature, high salt, H_2_O_2_, and drought (Li et al., 2013)
*Pisum sativum* (Common pea)	PsTCTP AAB19090	Cell division (Woo and Hawes, 1997)

## Materials and methods

### TCTP sequences

Protein sequences used in this analysis were retrieved from Phytozome database (http://www.phytozome.net/). As a starting point, the virtual translation derived from the nucleotide sequence of *Cucurbita maxima* TCTP (CmTCTP; Genbank accession number DQ304537) was obtained from the GenBank database (http://www.ncbi.nlm.nih.gov/nuccore/). Afterwards, a BLAST search was carried out using the corresponding CmTCTP protein sequence as query into the proteome Phytozome database. Based on this search, sequences representative of different plant species were selected for further study. Sequences that diverged from the consensus and were thus probably sequencing artifacts were not used for further analysis.

### Identity and similarity of TCTP protein sequences

The selected protein sequences of TCTP from different organisms were entered into MatGAT software version 2.01 (Matrix Global Alignment Tool) to calculate sequence similarity and identity (http://bitincka.com/ledion/matgat/). The series of pairwise alignments (shown as distance matrix) were inferred using the BLOSUM62 scoring matrix and the following parameters: number of first gap 10, gap extension 2. The analysis involved 59 amino acid sequences. Values were expressed as percent of similarity and identity between the TCTP protein sequences from different plant species compared with AtTCTP1 (At3g16640) from *A. thaliana* and CmTCTP from *C. maxima*.

### Phylogenetic analysis

The amino acid sequences chosen previously were aligned with the MUSCLE program (http://www.ebi.ac.uk/Tools/msa/muscle) using the profile method (Edgar, 2004). ProtTest program version 2.4 (Abascal et al., 2005) was used for the selection of the model of protein evolution that best fitted the set of sequences. Evolutionary analysis was conducted in PhyML 3.0 (Guindon et al., 2010) and the Molecular Evolutionary Genetics Analysis Program (MEGA) version 5 (Tamura et al., 2014). The evolutionary phylogenetic reconstruction (shown as dendrogram) was inferred using the LG+G evolution method and the following parameters: number of substitution rate categories 4, gamma shape parameter 0.642, and 1000 replicate bootstrap search with heuristic search options. Branches with <60% bootstrap support were collapsed. The tree was rooted with the *Cyanidioschyzon merolae* TCTP sequence, which belongs to the red algae group. The analysis involved 59 amino acid sequences.

### Predicted tertiary structure of TCTP

Full-length protein sequences were selected from the Phytozome database (http://phytozome.net) and refined through a comparison with the NCBI database (http://www.ncbi.nlm.nih.gov). Amino acid sequences were submitted to the automated protein structure homology-modeling program server SWISS-MODEL (http://swissmodel.expasy.org/), this server builds a model for each protein target using as templates, homologous protein structures which have been experimentally determined (Arnold et al., 2006; Kiefer et al., 2009). Swiss PDB Viewer application (http://www.expasy.org/spdbv/) was used for visualizing predictive 3D structures (Guex and Peitsch, 1997).

### Promoter analysis

Upstream sequences (1.0 kbp) from TCTP genes were retrieved from the Phytozome database (http://www.phytozome.net/). The list of genes and the species from which these upstream regions were obtained is listed in Table S1. These were classified according to their structural similarity to either AtTCTP1 or CmTCTP proteins from Arabidopsis and pumpkin, respectively. Also, since several of these genes have not been characterized, their 5′UTRs have not been defined; in these cases, 1 kb sequences upstream of the start codon were analyzed.

Promoter analysis was performed using the MEME software (http://meme.sdsc.edu/meme/meme.html, Bailey et al., 2006) with the following settings: one or more occurrence, per sequence, and a 10 base motif length. Based on sequence analysis, using ALIGNACE, as well as visual inspection of the promoter sets, it was determined that CT/GA repeats were present more than once in the analyzed upstream sequences and, thus, the ZOOPS and OOPS models were not employed. As background model the shuffled sequences were used and also analyzed with MEME (http://www.bioinformatics.org/sms2/about.html). Motifs were graphically represented with the WEBLOGO program (http://weblogo.berkeley.edu/logo.cgi). As background models, the shuffled sequences were likewise analyzed using the MEME algorithm.

## Results

### Plants harbor variable numbers of TCTP sequences

A BLASTP search was carried out for each of the plant species for which its genome has been sequenced using the CmTCTP protein sequence as query, after which the corresponding TCTP sequences were retrieved. The list of 59 TCTP sequences from different plant species used in this study is shown (Table S1). Based on the TCTP amino acid sequences retrieved from Phytozome database, the species that have only a single *TCTP* gene are: *Linum usitatissimum, Cucurbita maxima, Prunus persica, Malus domestica, Fragaria vesca, Citrus sinensis, Solanum tuberosum, Solanum lycopersicum, Sorghum bicolor, Zea mays, Setaria italica, Panicum virgatum, Oryza sativa, Brachypodium distachyon, Selaginella moellendorffi, Coccomyxa subellipsoidea* and *Cyanidioschyzon merolae*. Species that harbor 2 *TCTP* gene copies are: *Ricinus communis, Medicago truncatula, Phaseolus vulgaris, Glycine max, Cucumis sativus, Cucumis melo, Arabidopsis thaliana, Arabidopsis lyrata, Brassica rapa, Thellungiella halophila, Citrus clementina, Citrullus lanatus, Vitis vinífera, Mimulus guttatus* and *Physcomitrella patens*. Finally, the species that harbors 3 *TCTP* genes are: *Manihot esculenta, Populus trichocarpa, Gossypium raimondii* and *Aquilegia coerulea* (Table 2).

**Table 2:** **Structural classification of TCTP from plants**.

**Organism**	**AtTCTP1-like**	**CmTCTP-like**
*C. subellipsoidea*	C-169_65285	
*C. meroleae*	CMQ113C	
*P. patens*		XP_001757363; XP_001758666
*S. moellendorffi*	179722	
*O. sativa*	Os11g43900	
*B. distachyon*	Bradi4g10920	
*S. bicolor*	XP_002453140	
*Z. mays*	GRMZM2G108474_T01	
*S. italica*	Si026772m	
*A. coerulea*	Aquca_003_00740	Aquca_017_00176; Aquca_035_00202
*P. virgatum*		Pavirv00039226m
*S. tuberosum*		PGSC0003DMT400063579
*S. lycopersicum*	Solyc01g099780.2	
*M. guttatus*		Migut.G00151; Migut.N02086
*V. vinífera*		GSVIVT01017723001; GSVIVT01031135001
*C. sinensis*	1.1g030941m	
*C. clementina*	Ciclev10006071m	Ciclev10002699m
*G. raimondii*	Gorai.005G060700	Gorai.007G300300; Gorai.013G126000
*T. halophila*	BAJ33998	10022380m
*B. rapa*	Bra022172	Bra001637; Bra021187
*A. lyrata*	XP_002885160	XP_002884515
*G. max*		Glyma10g29240; Glyma09g04950
*P. vulgaris*		Phvul.007G197200; Phvul.009G248700
*M. truncatula*	Medtr1g083350; Medtr6g071090	
*F. vesca*	mRNA06814.1-v1.0-hybrid	
*M. domestica*		MDP0000164046
*P. persica*	ppa009639m	
*C. sativus*	Cucsa.253020	Cucsa.181820
*C. melo*	Melo3c006670p1	Melo3C015297P1
*C. lanatus*	Cla021747	Cla005200
*C. maxima*	N.D.	ABC02401
*P. trichocarpa*	Potri.005G024800; Potri.008G226500	Potri.010G013400
*L. usitatissimum*	Lus10033959	
*R. communis*		29726.m004052; 30128.m008835
*M. esculenta*	cassava4.1_025245m	cassava4.1_017738m; cassava4.1_017756m
*C. rubella*	EOA33129.1	EOA31592.1
*C. papaya*		evm.model.supercontig_1597.1; evm.model.supercontig_327.3

*Modeling of predictive 3D protein structure for each plant TCTP was carried out (Figure S4) and structure comparison was assessed to classify each plant TCTP based on their predictive structural relationship to either Arabidopsis thaliana AtTCTP1 or the Cucurbita maxima CmTCTP*.

Pairwise BLAST is used to calculate similarity, but its limitations are that only two sequences may be analyzed at one time and percent of similarity/identity is based on local alignment instead of a global alignment. Matrix Global Alignment Tool (MatGAT) is a simple, easy to use similarity/identity matrix generator that calculates the similarity and identity between every pair of sequences in a given data set without requiring pre-alignment of the data. The program performs a series of pairwise alignments using the Myers and Miller global alignment algorithm, calculates similarity and identity, and then places the results in a distance matrix (Campanella et al., 2003). Sequences were aligned with Arabidopsis AtTCTP1 and CmTCTP as type sequences, because biological function of the first one is better known, while the latter, albeit less well studied, appears to have a different function according to results in our group (see below; Hinojosa-Moya et al., 2013; Toscano-Morales et al., submitted).

Sequence comparison at the amino acid level of TCTPs from different species showed a similarity in the range of 80.4–98.2 and 82.1–94% compared with At3g16640 (AtTCTP1) and CmTCTP, respectively (Table S2). On the other hand, the percent of identities ranged from 70.2–95.2 to 71.2–88.1 with the reference AtTCTP1 and CmTCTP sequences respectively. These ranges did not include the percent of similarities/identities with the microalgae *C. subellipsoidea* and the red algae *C. merolae*.

### Phylogeny of plant TCTP protein sequences cannot be resolved satisfactorily

Protein sequences used in this analysis were retrieved from the Phytozome database. The amino acid sequences chosen were aligned with the MUSCLE program (Figure S1). A phylogeny of the aforementioned sequences was generated by MEGA 5.0 software by using the LG+G method with 1000 bootstrap replications. Phylogenetic analysis based on the TCTP protein sequence from different organisms groups may be informative with respect to the evolution of TCTP *per se*. But this also depends on which outgroup was used to root the tree. If this consists of a protein with a similar function, the tree may reflect the functional similarities between these proteins. On the other hand, an outgroup consisting of a sequence derived from an early branching event may yield more information on the phylogenetic relationship between the taxa harboring this protein (Hinojosa-Moya et al., 2008). For this analysis, TCTP sequence from the red algae *C. merolae* was used as outgroup. However, the resulting tree could not be resolved satisfactorily (Figure S2).

### Predictive protein structure of plant TCTPs as a parameter to establish potential functional differences

Since phylogenetic analysis did not provide sufficient information to suggest functional differences between plant TCTP sequences could be inferred; thus, other criteria were used to help establish such differences, if any. We had previously reported that while TCTP structures are highly conserved, there are subtle differences among some members of this family. Indeed, TCTP from *Plasmodium falciparum* and *P. knowlesi* harbor an extra α-helix close to the N terminus, not found in other TCTPs (Hinojosa-Moya et al., 2008); this causes a structural distortion in the pocket region that may be involved in the interaction with GTPases. We have recently found that such structural differences are reflected in the capacity of human and *P. falciparum* TCTP to induce B cell proliferation, as well as its incorporation into these cells (Calderón-Pérez et al., 2014). Thus, we assumed that plant TCTPs could also show structural differences from which functional specialization could be deduced. Therefore, TCTP amino acid sequences from each plant species whose genome has been sequenced were obtained from the Phytozome database, followed by direct comparison to sequences from other databases in order to avoid ESTs or repeated sequences. A total of 59 amino acid sequences were submitted to the automated protein structure homology-modeling server SWISS-MODEL (http://swissmodel.expasy.org/) that generated a homology model for each sequence. The respective model was visualized and modified to generate the 3D-models using the Swiss-Pdviewer 4.10 application.

The Arabidopsis genome harbors two *TCTP* genes, *AtTCTP1* and *AtTCTP2* (At3g16640 and At3g05540, respectively). The important role of the former in general growth and development has been described before. We have reported that the pumpkin (*Cucurbita maxima*) TCTP (CmTCTP) induces cell proliferation and regeneration, in conjunction with *A. rhizogenes rol* genes, and is phloem-mobile; thus, it can be considered that it is not a functional homolog of AtTCTP1 (Hinojosa-Moya et al., 2013). These results suggest that all TCTPs fall within these two broad groups, one of them related to AtTCTP1, while the other one more related to CmTCTP.

AtTCTP1 and CmTCTP were used as reference structures to determine whether this “division of labor” can be extrapolated to other plant species. It may be assumed that such functional specialization occurs in plants harboring more than one TCTP gene, while in those harboring a single TCTP gene this would perform the combined functions of AtTCTP1 and CmTCTP. Finally this specialization would be expected only in land plants, and especially in vascular plants.

**Figure 2:**
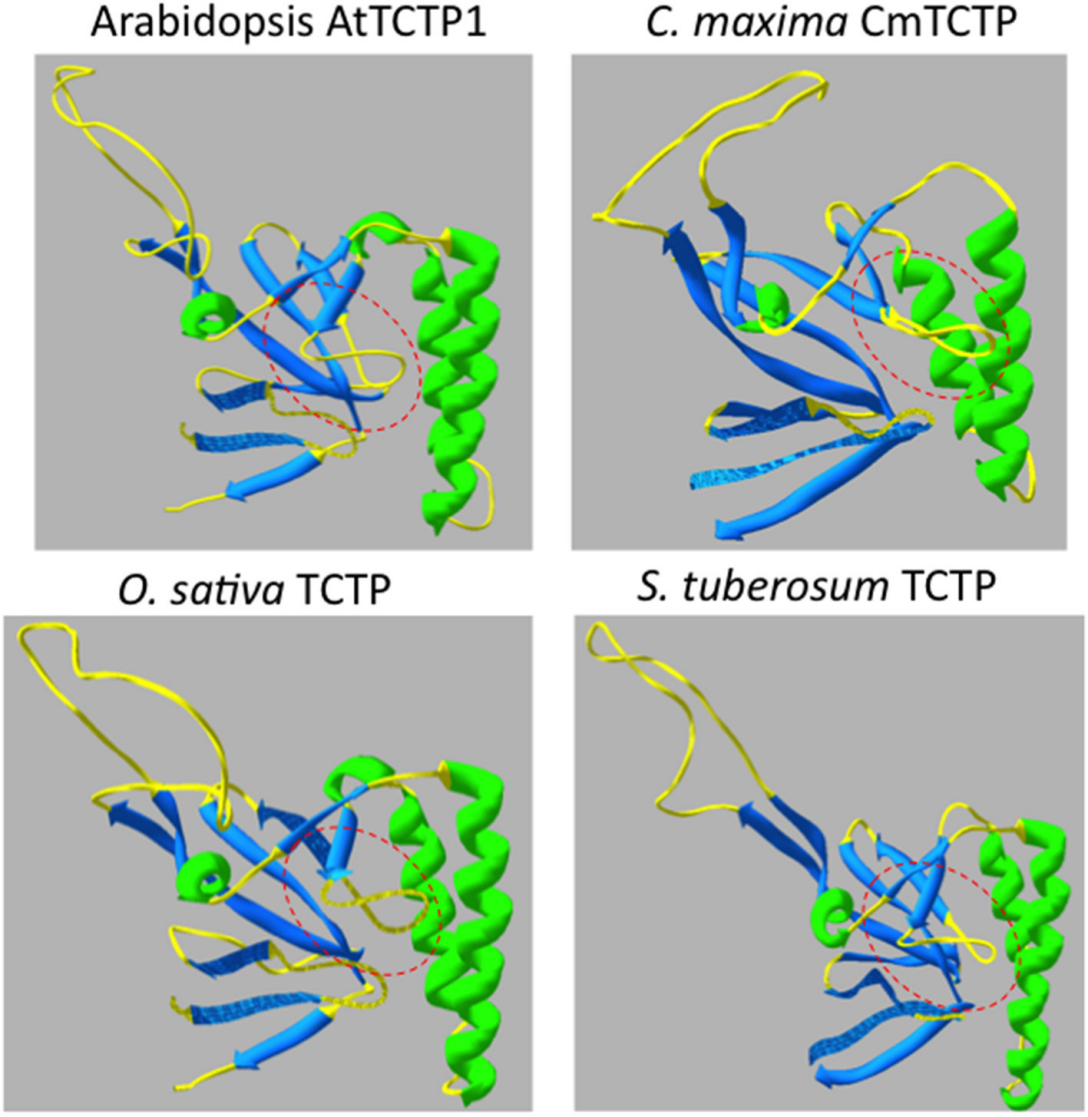
**Structural comparison of Arabidopsis TCTPs**. The “pocket” structure (highlighted in dashed red lines) and the flexible loop orientation indicate probable structural differences between these two TCTPs. In the bottom there are two examples of the structural classification made for all Plant TCTPs, retrieved from Phytozome database (http://phytozome.net). *O. sativa* TCTP (OsTCTP) is structurally related to AtTCTP1 while *S. tuberosum* (StTCTP) is predicted to be structurally similar to CmTCTP.

The 3D-predictive structures of all TCTPs were generated using Swiss Model; the templates used to obtain such structures are listed in Table S3. These structures presented a quite similar conformation. However, we found the “pocket” region near the center of the protein (and which may be the GTPase-binding domain, at least in fungi and animals) and its flexible loop as the most divergent regions among plant TCTPs. Interestingly, AtTCTP1 and CmTCTP meet this criterion of divergence. Indeed, the latter is predicted to be structurally similar to a group of TCTPs including StTCTP and FvTCTP, while AtTCTP1 is more related to other TCTP versions such as *Oryza sativa* TCTP (OsTCTP) (Figure 2). The predictive structures of diverse TCTPs support the notion that these proteins fall into two groups, AtTCTP1-like and CmTCTP-like (Table 2; Figures S3, S4), and based on these their possible functions can be inferred.

Intriguingly, we found no *TCTP* genes in several chlorophytes, e.g., *Ostreococcus lucimarinus, Micromonas pusilla, Volvox carteri*, and *Chlamydomonas reinhardtii*, with the exception of *Coccomyxa subellipsoidea*, which harbors one *TCTP* gene. This suggests that other proteins perform the roles played by TCTP in most plants are regulated through alternative routes. In contrast, the red algae *Cyanidioschyzon meroleae* harbors one *TCTP* gene.

The moss *Physcomitrella patens* harbors two *TCTP* genes, which encode a protein structurally similar to AtTCTP1 and the other resembling CmTCTP. In contrast, the tracheophyte *Selaginella moellendorffi* and several monocots (such as *Brachypodium dystachion, Oryza sativa, Sorghum bicolor, Zea mays, Setaria italic*, and *Panicum virgatum*) have only one gene coding for TCTP (all similar to AtTCTP1). Several eudicots harbor only one gene, but others contain two or more *TCTP* genes (Table 2). In the cases in which species have only one *TCTP* gene, this is usually *AtTCTP1*-like, as mentioned previously; but in some cases it resembles more *CmTCTP* (as in *G. max, P. vulgaris*, and *M. domestica*). Since the complete genome of pumpkin is not yet available, it is possible that it harbors an additional *TCTP* gene, which on a speculative note would be *AtTCTP1*-like protein. Indeed, considering that *C. melo* and *C. sativus* also harbor two copies of a *TCTP* gene, an *AtTCTP1*-like and a *CmTCTP*-like gene in each case, this is likely.

### *AtTCTP1*-like and *CmTCTP*-like upstream regions show different motif frequencies

**Figure 3:**
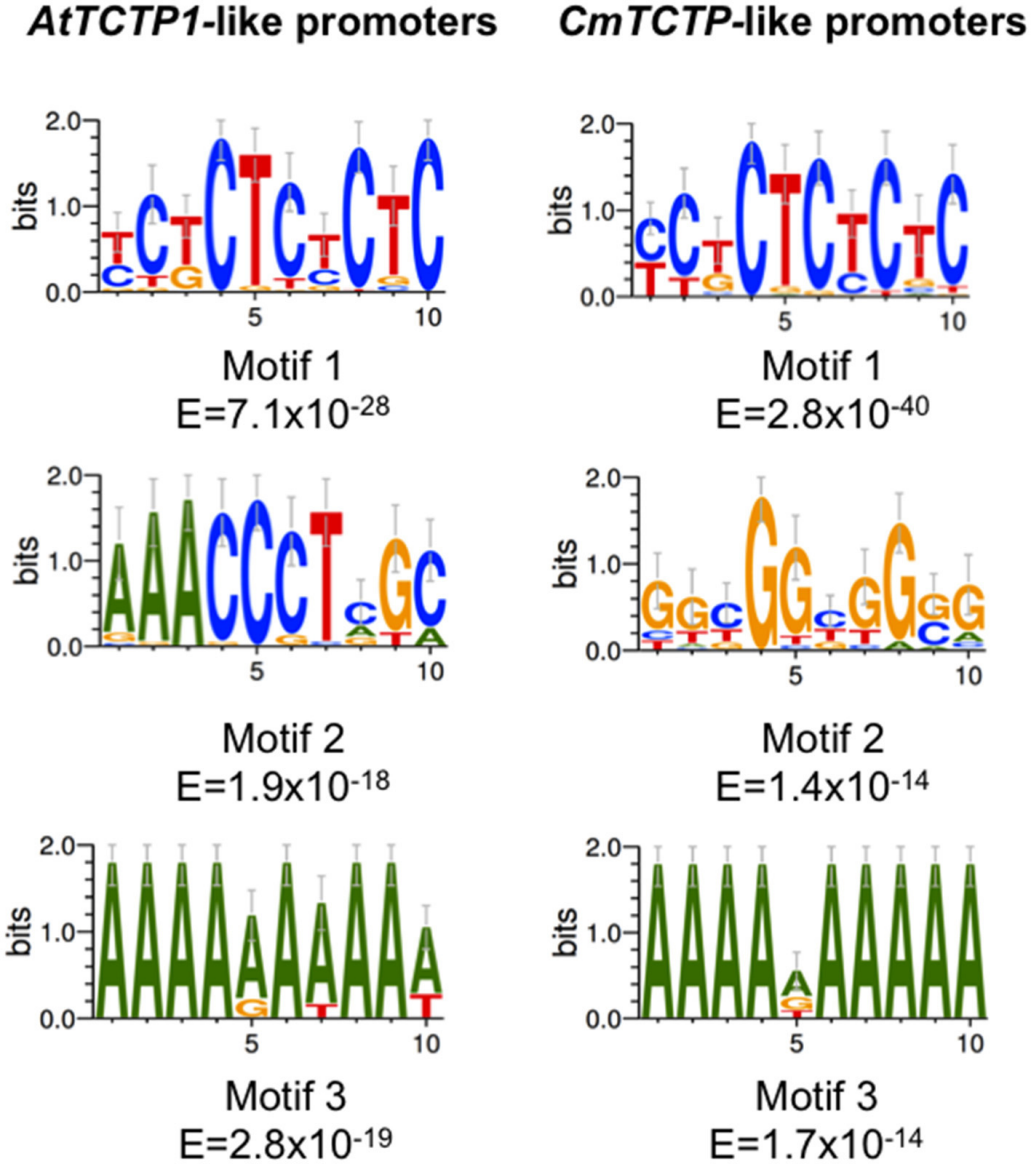
**MEME analyses of the TCTP promoters**. Consensus sequences identified in the promoters regions for both AtTCTP1-like and CmTCTP-like. Only the three motifs having the lowest *E*-values are shown.

Bioinformatic analyses of the upstream sequences from *TCTP* genes in plants identified common motifs in their sequences. Both sets of genes display overrepresented CT/GA motifs (motif 1) with an extremely low E value, indicating that these sets are expressed in vascular and/or rapidly growing tissues (Ruiz-Medrano et al., 2011; Figure 3). However, since the E value for the *CmTCTP-like* gene promoters is 12 orders of magnitude lower than for the corresponding *AtTCTP1*-like gene promoters, it is possible that there are differences in the expression levels or in their tissue-specific expression. On the other hand, the *AtTCTP1*-like promoters motif 2 is quite different from the corresponding motif 2 from the *CmTCTP*-like promoters, the former being an AC-rich element with a lower E value. Both gene promoters motif 3 sets is an A-rich element, quite common in upstream regulatory sequences. The shuffled sequences showed overrepresented motifs with very high (i.e., non-significant) *E*-values (10^1^–10^6^). These results suggest that the expression profiles of *AtTCTP1*-like and *CmTCTP*-like gene promoters may not overlap completely.

## Discussion

The known TCTP interactome indicates a wide variety of activities such as microtubule organization, inhibition of cell death, translation and ribosome function, DNA processing and repair, and modulation of the immune response, among several others (Nagano-Ito and Ichikawa, 2012; Amson et al., 2013). This translates to roles in general growth and developmental regulation, which in plants are less well known. Plant TCTPs show identities at the amino acid in the range of 70.2–95.2% and similarities of 80.4–98.2%, which support the notion of highly conserved functions for these proteins (See Table S2).

**Figure 4:**
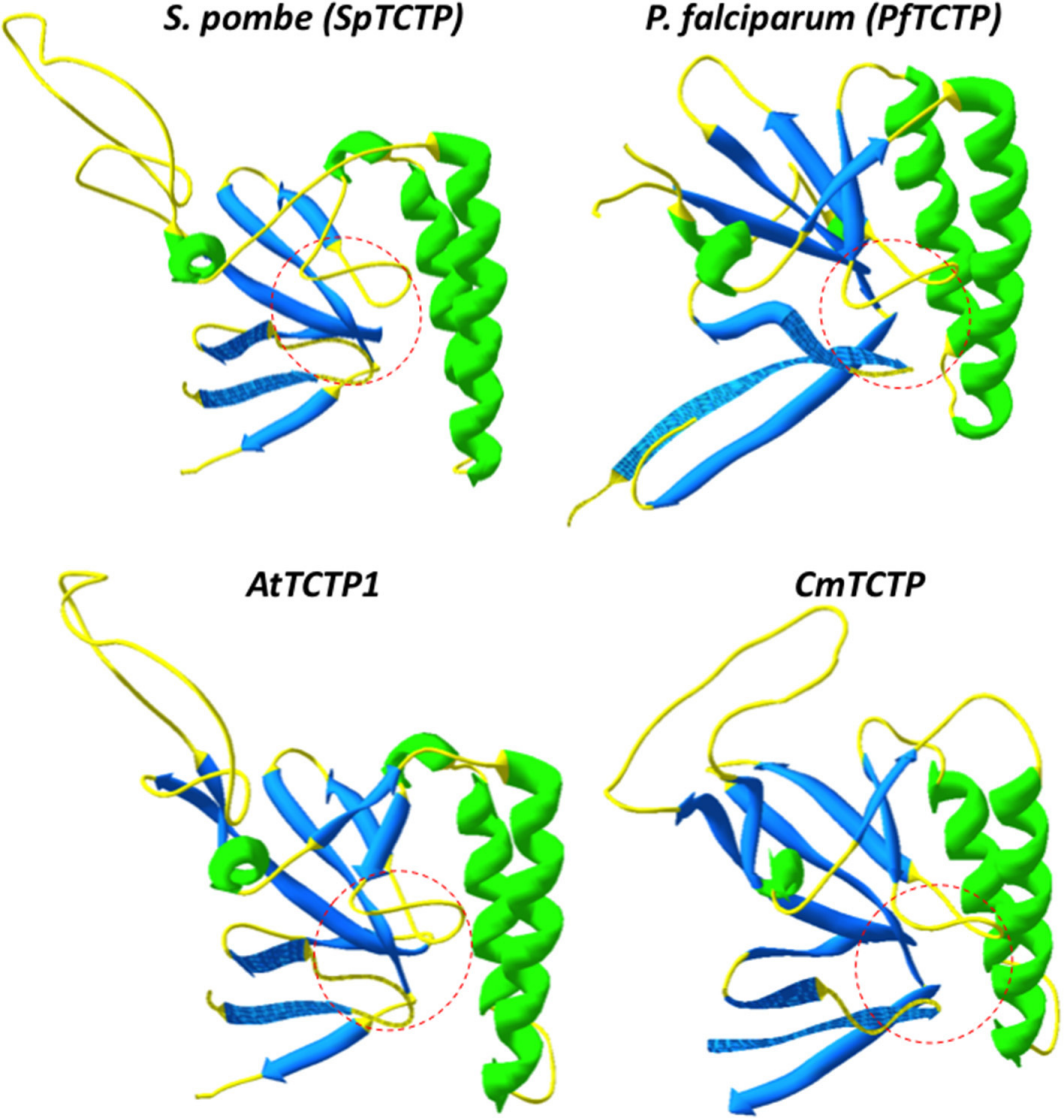
**Structural comparison between Arabidopsis and *Plasmodium falciparum* TCTPs**. The “pocket” structure (highlighted in dashed red lines) and the flexible loop orientation indicate probable structural differences between these TCTPs. As can be observed, the pocket region of AtTCTP1 and *S. pombe* TCTP (SpTCTP) have a “closed” conformation, while in CmTCTP and *P. falciparum* TCTP (PfTCTP) have an “open” conformation, that may cause altered interaction with its potential targets, or with different factors.

However, because of all these activities it is not clear whether TCTP is a multifunctional protein or if several closely related sequences have subtly different biochemical properties; this is especially relevant in cases where there is more than one gene per genome. For example, in humans, there are four TCTP genes, one of them corresponding to the histamine release factor (HRF), which is secreted. It is not known if this is the only TCTP that is secreted. Similarly, it is not clear if these genes display a common expression pattern. It is reasonable to assume that there is functional specialization among them. Another general assumption is that these proteins are structurally similar; however, our group has found that in some particular cases subtle structural differences underlie functional variations in TCTP. Indeed, the *P. falciparum* and *P. knowlesi* TCTP harbor an additional α-helix in the N-terminal domain (amino acids 22–30), which introduces an overall distortion in its structure, particularly in the central pocket region which may be involved in the recognition and binding of GTPases such as guanine-nucleotide exchange factors (Hsu et al., 2007; Hinojosa-Moya et al., 2008; Calderón-Pérez et al., 2014). It must be mentioned that the structures of the *P. falciparum* and *P. knowlesi* TCTPs have been experimentally determined (Vedadi et al., 2007; Eichhorn et al., 2013). Although this last activity has not been shown *in vitro*, it is clear that this domain is important for TCTP function. The *P. falciparum* TCTP is secreted into its host bloodstream, and can reach high titers, albeit its role in pathogenicity is not clear (MacDonald et al., 2001; Pelleau et al., 2012). Since HRF induces B cell proliferation, we suggested that the *P. falciparum* TCTP, which also induces histamine release (MacDonald et al., 2001), may mimic such activity, although not as efficiently as its host counterpart (Hinojosa-Moya et al., 2008). We have shown that *P. falciparum* TCTP induces B cell proliferation at low levels, but shows a different localization pattern when internalized into B cells (Calderón-Pérez et al., 2014), indicating that its structural distortion leads to a novel activity (possibly interfering with the host immune response). Interestingly, the structural variation of *Plasmodium* TCTPs was also observed in the CmTCTP-like proteins from plants, suggesting also functional variation relative to AtTCTP1-like proteins. Indeed, the pocket region, which is formed almost invariably by glutamic acid residues at positions 12 and 134 and leucine at 74 (using the numbering of residues from the *Schizosaccharomyces pombe* TCTP as reference) has an “open” conformation in most non-plant TCTPs and AtTCTP1-like proteins, while it displays a “closed” conformation in *P. falciparum* and *P. knowlesi* TCTPs, as well as in CmTCTP-like proteins (Figure 4). Interestingly, there are a few extant non-plant TCTP sequences that have substitutions in one of these three conserved residues, but no such substitutions were found in plant sequences. As with the *Plasmodium* genus, the AtTCTP1-like and CmTCTP-like proteins may display different biological activities, at least in the case of the type members of these groups in Arabidopsis and in pumpkin (Hinojosa-Moya et al., 2013; Toscano-Morales et al., submitted). Indeed, *CmTCTP* (and, intriguingly, *AtTCTP2*), but not *AtTCTP1* are capable of inducing whole-plant regeneration when harbored by *A. rhizogenes* (although not by *A. tumefaciens*, suggesting the involvement of *rol* genes and/or auxins in this process). It has been shown that AtTCTP1 has a central role in the regulation of cell proliferation, although not postmitotic growth (Berkowitz et al., 2008; Brioudes et al., 2010). This could be extrapolated to all AtTCTP1-like proteins, while CmTCTP-like proteins could have a more direct role in developmental reprogramming.

The phylogenetic relationship of plant TCTPs showed a large polytomy; thus, it could not be resolved satisfactorily. According to a previous analysis, in the cladogram of plant and non-plant TCTPs, two polytomies were likewise observed within the plant and vertebrate clades. In the case of plants, there were two groups, one of which being highly structured and reflecting the known phylogeny of angiosperms. However, the other group, whose phylogeny was not resolved, included sequences from both dicots and monocots in a polytomy (Hinojosa-Moya et al., 2008). It is generally accepted that phylogenies based on a single gene or protein sequence are prone to artifacts and tend to be inaccurate due to uneven rates of substitution. As shown in this and previous studies, this is the case for TCTP in plants, at least with the sequences used for the phylogenetic analysis. As substitution rates vary among members of the same lineage, as is suggested in plants, the data reinforce possible differential selective pressure on TCTP sequences. We expected that using a TCTP sequence from a phylogenetically distant organism (a red alga, *C. merolae*) as outgroup to root the tree could reflect the functional similarities between these proteins; nevertheless, phylogenetic relationships could not be established either. Low bootstrap values underscore the high similarity of plant TCTP sequences.

### Emergence of *CmTCTP*-like genes and different role of the corresponding proteins

In general terms, it appears that *CmTCTP*-like genes could arise roughly during the emergence of land plants through gene duplication of an ancestral *AtTCTP1*-like gene (which is deduced from the nature of the extant TCTP sequence from *C. subellipsoidea*). The absence of this gene in most chlorophyte genomes could be due to technical problems, explained if it is located close to centromeres or telomeres, which are regions notoriously difficult to sequence. However, if these genes are really not present, then other proteins must perform the function carried out by TCTP, the nature of which is not clear. The loss of *TCTP* genes in these lineages cannot be discarded either, but a more thorough analysis of the regions flanking the TCTP gene in *C. subellipsoidea* and the homologous regions in other chlorophytes may help elucidate this question. In any case, the plant TCTP interactome could help elucidate the pathways regulated by the proteins that replaced them in these species, such as regulation of cell cycle duration and proliferation (Brioudes et al., 2010). In the case of AtTCTP2, it arose recently, before the divergence of the *Arabidopsis* genus; indeed, the position of the *A. lyrata AtTCTP2* homolog in the genome is equivalent to its Arabidopsis counterpart. AtTCTP1 and AtTCTP2 show an identity superior to 80%; the most visible difference is a 13 aa deletion of AtTCTP2 relative to AtTCTP1. No other TCTP harbors such deletion, except *Giardia lamblia* TCTP. Interestingly, the deletion falls in the same region (the flexible loop) as in AtTCTP2; this loop may be an hypervariable region that in all predicted TCTP structures falls outside of the domains known to be involved in some of its activities (Ca^+2^ and microtubule binding for example). However, it appears that distortions in such domain may alter the overall conformation of these proteins and hence their function.

The analysis of the predictive 3D conformation shows that it is feasible to infer that there may be a division of the labor among plants harboring multiple TCTP genes, and moreover that in some cases the unique TCTP version could perform several functions. Clearly, experimental evidence must be obtained in order to test this hypothesis; however, this can be done in a straightforward manner; if this is correct, all *AtTCTP1*-like genes will be unable to regenerate tobacco plants via *A. rhizogenes* mediated inoculation of leaf explants, in contrast to *CmTCTP*-like genes (Hinojosa-Moya et al., 2013; Toscano-Morales et al., submitted). Given that the same underlying mechanisms of cell proliferation operate in plants and other eukaryotes, this simple assay could be used to determine whether a TCTP gene is involved in cell proliferation or cell (developmental) reprogramming.

## Concluding remarks

The exact mechanism through which TCTP regulates plant growth and development is not clear. The fact that there may be at least two different groups of TCTP genes in plants, one more involved in the regulation of cell proliferation, and the other one in cell reprogramming, was inferred from the activity of two proposed representative members of this protein family in plants, AtTCTP1 and CmTCTP from Arabidopsis and *C. maxima*, respectively. On the other hand, such differential activity correlates with subtle structural variations of the predicted three-dimensional structures of these proteins. Interestingly, these structural variations are also present in other eukaryotes, which may also correlate with functional variation. We propose that most plant TCTPs fall within one of these groups, and that a simple assay, i.e., regeneration of tobacco when harbored by *A. rhizogenes*, could help determine a role in cell proliferation alone or cell reprogramming.

### Conflict of interest statement

The authors declare that the research was conducted in the absence of any commercial or financial relationships that could be construed as a potential conflict of interest.

## Supplementary material

Figure S1 **Protein sequence alignment**. The amino acid sequence alignment was performed using the MUSCLE software on plant sequences included in this work.

Figure S2 **Phylogenetic analysis of the TCTP in plants**. *C. maxima* translationally controlled tumor protein (TCTP) sequence (GenBank Accession No. ABC02401) was retrieved form NCBI (http://www.ncbi.nlm.nih.gov) and used as query. Protein homologs were identified by BLAST search against the phytozome database (http://phytozome.net). Full-length protein sequences were aligned with MUSCLE (http://www.ebi.ac.uk/Tools/msa/muscle). ProtTest program (PROTTEST 2.4, Abascal et al., 2005) was used for the selection of the model of protein evolution that best fitted the set of sequences. Evolutionary analysis was conducted in PhyML 3.0 (Guindon et al., 2010) and MEGA5. The evolutionary phylogenetic reconstruction (shown as dendrogram) was inferred using the LG+G evolution method and the following parameters: number of substitution rate categories 4, gamma shape parameter 0.642. Bootstrap values higher than 60% are shown (1000 replicates). The analysis involved 55 amino acid sequences.

Figure S3 **Predictive 3D structure comparison of AtTCTP-like proteins**. Full-length protein sequences were selected from phytozome database (http://phytozome.net) and refined through a comparison with NCBI database (http://www.ncbi.nlm.nih.gov). Amino acid sequences were submitted to the automated protein structure homology-modeling program server SWISS-MODEL (http://swissmodel.expasy.org/), this server builds a model for each protein target using as templates homologous protein structures which have been experimentally proved (Arnold et al., 2006; Kiefer et al., 2009). Swiss PDB Viewer application (http://www.expasy.org/spdbv/) was used for visualizing predictive 3D structures (Guex and Peitsch, 1997).

Figure S4 **Predictive 3D structure comparison of CmTCTP-like proteins**. Full-length protein sequences were selected from phytozome database (http://phytozome.net). Amino acid sequences were submitted to the automated protein structure homology-modeling program server SWISS-MODEL. Swiss PDB Viewer application (http://www.expasy.org/spdbv/) was used for visualizing predictive 3D structures (Guex and Peitsch, 1997).
