# Supplementary material for: Structural divergence of plant TCTPs
Source: Front Plant Sci. 2014 Jul 29;5:361. doi: 10.3389/fpls.2014.00361 (PMC4114181; doi:10.3389/fpls.2014.00361)
Supplement: Figure S1 — Protein sequence alignment. The amino acid sequence alignment was performed using the MUSCLE software on plant sequences included in this work. [file DataSheet1.DOC]

Figure S1

Gutiérrez-Galeano *et al*., 2014

CLUSTAL 2.1 multiple sequence alignment

C.sativ_Cucsa.181820_ MLLYQDLLTGDELLSDSFP-YNEIENGMLWEVEGKWVVQGAIDVD--IGA 47

C.melo_MELO3C015297P1__uniprot MLLYQDLLTGDELLSDSFP-YNEIENGMLWEVEGKWVVQGAIDVD--IGA 47

C.lunata_Cla005200 MLVYQDLLTGDELLSDSFP-YKEIENGMLWEVEGKWVVQGAIDVD--IGA 47

G.max_Glyma10g29240_ MLVYQDLLTGDELLSDSFP-YKETENGMLWEVEGKWVVQGAVNVD--IGA 47

R.communis_30128.m008835_ MLVYQDLLTGDELLSDSFP-YKEIQNGMLWEVEGKVVVQGAIDVD--IGA 47

M.esculenta_cassava4.1_017738m MLVYQDLLTGDELLSDSFP-CKEIENGMLLEVEGKWVVQGAIDVD--IGA 47

V.vinifera_GSVIVT01031135001_ MLVYQDLLTGDELLSDSFP-YKEIENGMLWEVEGKWVVQGAVDVD--IGA 47

M.truncatula_Medtr1g083350_ MLVYQDLLTGDELLSDSFP-YKEIENGMLWEVEGKWVVQGAVDVN--IGA 47

P.vulgaris_Phvul.007G197200_ MLVYQDLLTGDELLSDSFP-YKEIENGMLWEVEGKWVVQGAVNVD--IGA 47

M.truncatula_Medtr6g071090_ MLVYKDLLIGDELLSDSYP-YKEIDNGMLSEVEGKWVVRGACDVD--IGA 47

M.esculenta_cassava4.1_017756m MLVYQDLLTGDELLSDAFP-YKEIENGMLWEVQGKWVVKGAVDVD--IGA 47

A.thaliana_At3g05540_AAF26143_ MLVYQDILTGDELLSDSFP-YKEIENGMLWEVEGKWVVKGAMDFD--IGA 47

A.lyrata_XP_002884515_ MLVYQDILTGDELLSDSFP-YKEIENGMLWEVEGK--------------- 34

T.halophila_10022380m_ MLVYQDLLTGDELLSDSFP-YKEIENGMLWEVEGKWVVKGAMNFD--IGA 47

G.raimondii_Gorai.013G26000_ MLVYQDILTGDELLSDSFP-YKEIENGMLWEVEGKWVVQGAVTVD--IGA 47

G.raimondii_Gorai.007G300300_ MLVYQDLLTGDELLSDSFP-YKEIENGMLWEVEGKWVVQGAVNVD--IGA 47

C.clementina_Ciclev10002699m_ MIVYQDLLSGDELLSDSFP-YKEIENGMLWEVEGKWVVQGAVNVD--IGA 47

C.sinensis_1.1g030941m_ MLVYQDLLTGDELLSDSFP-YKEIENGILWEVEGKWVVQGAVDVD--IGA 47

C.clementina_Ciclev10006071m_ MLVYQDLLTGDELLSDSFP-YKEIENGILWEVEGKWVVQGAVDVD--IGA 47

M.esculenta_cassava4.1_025245m MLLYQDLLTGDELLSDSFS-YKEIHNGMLWEVEGKWVVQGAVDVD--IGA 47

L.usitatissimum_Lus10033959_ MLVYEDLVTGDELLSDSFP-YNEILDGALWEVEGKWVVQGAVDVN--IGA 47

P.trichocarpa_Potri.010G013400 MLVYQDLLSGDELLSDSFP-YKEIENGILWEVEGKWVVQGAVDVD--IGA 47

P.trichocarpa_Potri.008G226500 MLVYQDLLSGDELLSDSFP-YKEIENGILWEAEGKWVVQGAVDVD--IGA 47

G.max_Glyma09g04950_ MLVYQDLLTGDELLSDSFR-YKEIENGMLWEVEGKWVVKGAVDVD--IGA 47

P.vulgaris_Phvul.009G248700_ MLVYQDLLTGDELLSDSFR-YTEIENGMLWEVEGKWVVQGAVEVN--TGA 47

G.raimondii_Gorai.005G060700_ MLVYQDLISGDELLSDSFS-YKEIENGMLWEVEGKWVVQGAIDVD--IGA 47

S.bicolor_XP_002453140_ MLVYQDLLSGDELLSDSFQ-YKEIFDGVLWEVEGKWVVKGAVDVD--IGA 47

P.virgatum_Pavirv00039226m_ MLVYQDLLTGDELLSDSFP-YKEIENGVLWEVEGKWVVQGAVDVD--TGA 47

S.italica_Si026772m_ MLVYQDLLTGDELLSDSFP-YREIENGILWEVDGRWVVQGAVDVD--IGA 47

O.sativa_Os11g43900_ MLVYQDLLTGDELLSDSFP-YREIENGILWEVDGKWVVQGAIDVD--IGA 47

M.domestica_MDP0000164046_ MLVYQDLLTGDELLSDSFP-YREIHNGVLWEVDGKWVVQGAVDVD--IGA 47

P.persica_ppa009639m_ MLVYQDLLTGDELLSDSFP-YKEVHNGVLWEVDGKWVVQGAVDVD--IGA 47

F.vesca_mrna06814.1-v1.0-hybri MLVYQDLLSGDELLSDSFP-YKEIQNGVLWEVDGKWVVQGAVDVD--IGA 47

V.vinifera_GSVIVT01017723001_ MLVYQDLLTADELLSDSFP-YKELFNGALWEVEGKWVVQGAIDVD--IGA 47

R.communis_29726.m004052_ MLVYQDLLTGDELLSDSFP-YKEIENGILWEVEGKWVVQGAIDVD--IGA 47

S.tuberosum_PGSC0003DMT4000635 MLVYQDLLTGDELLSDSFP-YKEIQNGMLWEVQGKWVVQGAVDVN--IGA 47

S.lycopersicum_Solyc01g099780_ MLVYQDLLTGDELLSDSFP-YKELENGMLWEVQGKWVVQGAVDVN--IGA 47

A.coerulea_Aquca_035_00202_ MLVYEDLLTGDELLSNSFP-YKKIENGILWEVEGKWVVQGAVNVD--IGV 47

A.coerulea_Aquca_003_00740_ MLVYEDLLTGDELLSDSFP-YEEIENGILWEVEGKWVVQGAIDVD--IGA 47

C.sativus_Cucsa.253020_ MLVYQDLVSGDELLSDSFP-YKEIENGMIWEVEGKWVVKGAVDVD--IGA 47

C.melo_MELO3c006670p1__uniprot MLVYQDLVSGDELLSDSFP-YKEIENGMIWEVEGKWVVKGAVDVD--IGA 47

C.maxima_ABC02401_ MLVYQDLLTGDELLSDSFP-YKELENGMIWEVEGKWVVQGAVDVD--IGA 47

C.lunata_Cla021747 MLVYTDLLSGDELLSDSFP-YKEIENGMIWEVDGKWVVQGAIDVD--IGA 47

A.thaliana_At3g16640_AAM66134_ MLVYQDLLTGDELLSDSFP-YKEIENGILWEVEGKWVTVGAVDVN--IGA 47

B.rapa_Bra022172_ MLVYQDLLTGDELLSDSFP-YKEIENGILWEVEGKWTTVGAVDVN--IGA 47

T.halophila_BAJ33998_ MLVYQDLLTGDELLSDSFP-YKEIENGILWEVEGKWVTVGAVDVN--IGA 47

B.rapa_Bra001637_ MLVYTDLLTGDELLSDSFP-YKEIENGILWEVEGKWTTKGCVEVN--IGA 47

A.lyrata_XP_002885160_ MLVYTDLLTGDELLSDSFP-YKEIENGILWEVEGKWVTLGAVDVN--IGA 47

P.trichocarpa_Potri.005G024800 MLVYQDLLTGDELLSDSFP-YKEIENGVLWEVEGKWVVQGSIDVD--IGA 47

A.coerulea_Aquca_017_00176_ MLVYQDLLTGDELLSDSFP-YKEIENGILWEVEGKWVVQGAVDVD--IGA 47

B.distachyon_Bradi4g10920_ MLVYQDKLTGDELLSDSFP-YKELENGVLWEVDGHWVVQGAVDVD--IGA 47

M.guttatus_Migut.N02086_ MLVYQDLLSGDELLSDSFP-YTEIENGMLWEVEGRWVVQGAVNVD--IGA 47

M.guttatus_Migut.G00151_ MLVYQDLLTGDELLSDSFP-YKEIENGALWEVEGKWVVTGSVDVD--IGA 47

P.patens_XP_001758666_ MLVYQDLISGDELLSDSFE-YKELFNGVLWEVEGKWVVKGALDVDALIGA 49

P.patens_XP_001757363_ MLVYQDLVSGDELLSDSFD-YKELFNGVLWEVEGKWVVKGALDVDALIGA 49

S.moellendorffi_179722_ MLVYQDLLSGDELLSDSFP-YKEIQNGVLWEVEGKWVVTGCVDVD--IGA 47

Z.mays_GRMZM2G108474_T01_ MLVYQDLLSGDELLSDSFT-YKELENGVLWEVEGKWVTQGPVDVD--IGA 47

C.subellipsoidea_C-169_65285_ MLIYKDLLSGDELISDSYN-MKESEDGFFYEVDGKWVTVGDVDVD--IGA 47

C.merolae_CMQ113C_ MIVWKDVFTKDELLSDGMKNVRELENGLLLACDSYNVTLGGGDYG--IAN 48

*::: * . ***:*:. : :* : :.

C.sativ_Cucsa.181820_ NPSAEGADEDEGVDDQAVKVVDIVDTFRLQEQPSFDKKQFITYMKRYIKL 97

C.melo_MELO3C015297P1__uniprot NPSAEGADEDEGVDDQAVKVVDIVDTFRLQEQPPFDKKQFITYMKRYIKL 97

C.lunata_Cla005200 NPSAEGAEDDEGVDDQAVKVVDIVDTFRLQEQPPFDKKQFITYMKRYIKL 97

G.max_Glyma10g29240_ NPSAEGGDEDEGVDDQAVKVVDIVDTFRLQEQPPFDKKQFITYMKRYIKL 97

R.communis_30128.m008835_ NPSAEGGGEDEGVDDQAVKVVDIVDTFRLQEQPSFDKKQFVTYMKRYIKL 97

M.esculenta_cassava4.1_017738m NPSAEGGGEDEGVDDQAVKVVDIVDTFRLQEQPSFDKKQFVTYMKRYIKL 97

V.vinifera_GSVIVT01031135001_ NPSAEGGGEDEGVEDQAVKVVDIVDTFRLQEQPSFDKKQFVTFMKRYIKL 97

M.truncatula_Medtr1g083350_ NPSAEGGDEDDGVDDQAVKVVDIVDTFRLQEQPTFDKKQFVTYMKRYIKL 97

P.vulgaris_Phvul.007G197200_ NPSAEGGEDDDGVDDQAVKVVDIVDTFRLQEQPSYDKKQFVTFIKRYIKL 97

M.truncatula_Medtr6g071090_ NPYAEGG-EDEGVDDSTAKVVDIVDVFRLQEQLAFDKKQFLGFVKRYIKL 96

M.esculenta_cassava4.1_017756m NPSAEGEKEDEGLDDPAVKVVDIVDTFRLREQFAFDKQQFLAFMKRYIKL 97

A.thaliana_At3g05540_AAF26143_ NP-GEEGGEDEGVDDQAVKVVDIIDTFRLQEQPSFDKKQFVMFMKRYIKQ 96

A.lyrata_XP_002884515_ NPSAEEGGEDEGVDDQAVKVVDIIDTFRLQEQPSFDKKQFVMFMKRYIKQ 84

T.halophila_10022380m_ NPSAEEGGEDEGVDDQAVKVVDIIDTFRLQEQPSFEKKQFVMFMKRYIKQ 97

G.raimondii_Gorai.013G26000_ NPSAEGGDEDEGVDDQAIKVVDIVDTFRLQEQPAFDKKQFVTYMKRYIKN 97

G.raimondii_Gorai.007G300300_ NPSAEGADDDEGVDDQAVKVVDIVDTFRLQEQPAFDKKQFVTFMKRYIKN 97

C.clementina_Ciclev10002699m_ NPSAEGGDDDEGVDDQAVKVVDIVDTFRLQEQPAFEKKQFVTFMKRYIKL 97

C.sinensis_1.1g030941m_ NPSAEGADEDEGVDDQAVKVVDIVDTFRLQEQPAFDKKQFVTYMKRFIKL 97

C.clementina_Ciclev10006071m_ NPSAEGADEDEGVDDQAVKVVDIVDTFRLQEQPAFDKKPFVTYMKRFIKL 97

M.esculenta_cassava4.1_025245m NPSAEGADEDEGVDDQAVKVVDIVDTFRLQEQPSFDKKQFVTYMKRFIKL 97

L.usitatissimum_Lus10033959_ NPSAEGADEDEGVDDQAAKVVDIVDTFRLQEQPAFDKKQFVTYIKRYIKA 97

P.trichocarpa_Potri.010G013400 NPSAEGGDEDEGVDDQAAKVVDIVDTFRLQEQPPFDKKQFLTQIKKFIKN 97

P.trichocarpa_Potri.008G226500 NPSAEGGDEDEGVDDQTVKVVDIVDTFRLQEQPAFDKKQFVTYMKRFIKL 97

G.max_Glyma09g04950_ NPSAEGGGEDEGVDDAAVKVVDIVDTFRLQEQPAFDKKQFVTFMKRFIKN 97

P.vulgaris_Phvul.009G248700_ NPSAEGG-DEEGVDDQAVKVVDIVDTFRLQEQPTFDKKAFVTYMKRFIKN 96

G.raimondii_Gorai.005G060700_ NPSAEDADEDEGVDDQSVKVVDIVDTFRLQEQPPFDKKQFVVFMKKFIKN 97

S.bicolor_XP_002453140_ NPSAEGG-EDEGVDDQTERVVDIVDTFRLQEQPTFDKKTFVTNIKRYIKN 96

P.virgatum_Pavirv00039226m_ NPSAEGG-EDEGVDDQAVKVVDIVDTFRLQEQPAFDKKQFVTFIKRYIKN 96

S.italica_Si026772m_ NPSAEGGGDDEGVDDQAVKVVDIVDTFRLQEQPAFDKKQFVTFMKRYIKN 97

O.sativa_Os11g43900_ NPSAEGGGDDEGVDDQAVKVVDIVDTFRLQEQPPFDKKQFVTFMKRYIKN 97

M.domestica_MDP0000164046_ NPSAXGGGEDEGVDDQAVKVVDIVDTFRLQEQPPYDKKQFVTWVKRYIKL 97

P.persica_ppa009639m_ NPSAEGGGEDEGVDDQTVKVVDIVDTFRLQEQPPFDKKQFVTWVKRYIKL 97

F.vesca_mrna06814.1-v1.0-hybri NPSAEGADGDEGVDDQTVKVVDIVDTFRLQEQPPFDKKQFVTWVKRYIKL 97

V.vinifera_GSVIVT01017723001_ NPSAEGG-EEEGVDDQTVKVVDIVDTFRLQEQPPFDKKQFVTYMKRYIKL 96

R.communis_29726.m004052_ NPSAEGGGEDEGVDDQAVKVVDIVDTFRLQEQPPFDKKQFVTFIKRYIKL 97

S.tuberosum_PGSC0003DMT4000635 NPSAEGGGEDEGVDDQAVKVVDIVDTFRLQEQPAFDKKQFVTYIKRYIKN 97

S.lycopersicum_Solyc01g099780_ NPSAEGGGEDEGVDDQAVKVVDIVDTFRLQEQPAFDKKQFVTYMKRYIKN 97

A.coerulea_Aquca_035_00202_ NPSAEGG-DDEGVDDQAVKVVDIVDTFRLQEQPSFDKKQFVTYMKRYIKL 96

A.coerulea_Aquca_003_00740_ NPSAEGG-DDEGVDDQAVKVVDIVDTFRLQEQPSFDKKQFVTYIKRYIKL 96

C.sativus_Cucsa.253020_ NPSAEGGGDDEGVDDQAVKVVDIVDTFRLQEQPSMDKKVFLTCIKKYIKK 97

C.melo_MELO3c006670p1__uniprot NPSAEGGGEDEGVDDQAVKVVDIVDTFRLQEQPSMDKKVFLSCIKKYIKK 97

C.maxima_ABC02401_ NPSAEGDGEDEGVDDQAVKVVDIVDTFRLQEQPTMDKKQFIAYIKKFIKL 97

C.lunata_Cla021747 NPSAEGAGEDEGVDDQAVKVVDIVDTFRLQEQPPLDKKLFIAYIKKYIKL 97

A.thaliana_At3g16640_AAM66134_ NPSAEEGGEDEGVDDSTQKVVDIVDTFRLQEQPTYDKKGFIAYIKKYIKL 97

B.rapa_Bra022172_ NPSAEEGGEDEGVDDTTQKVVDIVDTFRLQEQPTYDKKGFIAYIKKYIKL 97

T.halophila_BAJ33998_ NPSAEEGGEDEGVDDTTQKVVDIVDTFRLQEQPTYDKKGFIAYIKKYIKL 97

B.rapa_Bra001637_ NPSAEEGGEDEGVDDSVEKVVDIVDTFRLQEQPTYDKKGFIAYIKKYIKL 97

A.lyrata_XP_002885160_ NPSAEEGGEDEGVDDSAQKVVDIVDTFRLQEQPTYDKKGFVAYIKKYIKS 97

P.trichocarpa_Potri.005G024800 NPSAEGG-DDEGVDDQAVKVVDIVDTFRLQEQPAFDKKQFVTYIKRYIKL 96

A.coerulea_Aquca_017_00176_ NPSAEGGGEDEGVDDQAVKVVDIVDTFRLQEQPAFDKKQFVAFIKRFIKQ 97

B.distachyon_Bradi4g10920_ NPSAEGGGEDEGVDDQAVKVVDIVDTFRLQEQPAFDKKQFVAYIKRYIKN 97

M.guttatus_Migut.N02086_ NPSAEGGEEDEGVDDQSVKVVDIVDTFRLQEQPAFDKKQFVAYIKKYIKN 97

M.guttatus_Migut.G00151_ NPSAEGGGEDEGVDDQAVKVVDIVDTFRLQEQPPFDKKQFVAYIKKYIKS 97

P.patens_XP_001758666_ NASAEGGGEDEGVSDEAVKVVDIIDTFRLQEQPAFDKKTFMAYIKKYLKK 99

P.patens_XP_001757363_ NASAEGGGEDEGVNDEAVRVVDITDTFRLQEQPAFDKKGFTAFIKKYLKI 99

S.moellendorffi_179722_ NPSQEGGEDDEGVDDQAAKVVDIVDTFRLQEQPAFDKKTFLGCMKKFIKN 97

Z.mays_GRMZM2G108474_T01_ NPSAEGG-EDESVDDTAVKVVDIVDTFRLQEQPPFDKKSFVSYIKKYIKN 96

C.subellipsoidea_C-169_65285_ NPSAEEE--EDGVDSQSRKVVDVVDAFRLNEQPSYDKKQFLGWAKEWLKK 95

C.merolae_CMQ113C_ NDEEAMGAGGDTAGAESVNVV--VDAFKLQN-IQLPRKDFQVYIKKYSKR 95

* : .** *.*:*.: :: * *.: *

C.sativ_Cucsa.181820_ LTPKLDEEKQELFRKHIPEATKFLISKIDDL---QFFVGEGM-HDDGTMV 143

C.melo_MELO3C015297P1__uniprot LTPKLDEEKQELFKKYIPEATKFLISKINDL---QFFVGEGM-HDDGTMV 143

C.lunata_Cla005200 LTPKLDEEKQELFKKYIPEATKFLLSKINDL---QFFVGEGM-HDDGTTV 143

G.max_Glyma10g29240_ LTAKLEGEQQELFKKHIEGATKSLLSKLKDL---QFFVGESM-HDDGSLV 143

R.communis_30128.m008835_ LTAKLEPEKQELFKKNIEAATKFLLSKLSDL---QFFVGESM-HDDGSLV 143

M.esculenta_cassava4.1_017738m LTPKLEPEKQELFKKHIEGATKFLLAKLSDL---QFFVGESM-HDDGSLV 143

V.vinifera_GSVIVT01031135001_ LTPKLEPEKQELFKKHIEGATKFLLPKLSDL---QFFVGESM-HDDGSLV 143

M.truncatula_Medtr1g083350_ LTSKLEPEKQELFKKHIEAATKFLLPKLKDL---QFFVGESM-HDDGSLV 143

P.vulgaris_Phvul.007G197200_ LTPKLEAEKQELFKKHIEGATKFLLPKLKDL---QFFVGESM-HDDGSLV 143

M.truncatula_Medtr6g071090_ LIPKLDAAKQELFKKHIEGATKYLLGKLKDL---QFFVGESM-HDDGSLV 142

M.esculenta_cassava4.1_017756m LTPKLEPEKQESFKKHIEGATKFLLSKLEDL---QFFVGESM-HDDSSLV 143

A.thaliana_At3g05540_AAF26143_ LSPKLDSENQELFKKHIESATKFLMSKLKDF---QFFVGESMEGEEGSLV 143

A.lyrata_XP_002884515_ LSPKLDSEKQELFKKHIESATKFLMSKLKDF---QFFVGESMEGEEGSLV 131

T.halophila_10022380m_ LNPKLDSEKQELFKKHIESATKFLMSKLKDF---QFFVGESM-GEEGSLV 143

G.raimondii_Gorai.013G26000_ LAPKLEAAKQETFKKNIEGATKFLLAKLKDL---QFFVGESM-HDDGSLV 143

G.raimondii_Gorai.007G300300_ LTPKLEPEKQESFKKNIEGATKFLMSKLKDL---QFFVGESM-HDDGSLV 143

C.clementina_Ciclev10002699m_ LTPKLEAEKQELFKKNIEGATKFLLSKLSDL---QFFVGESM-QDDAGLV 143

C.sinensis_1.1g030941m_ LTPKLSEERQEIFKKNIEGATKFLLSKLSDL---QFFVGESM-HDDGCLV 143

C.clementina_Ciclev10006071m_ LTPKLSEERQEIFKKNIEGATKFLLSKLSDL---QFFVGESM-HDDGCLV 143

M.esculenta_cassava4.1_025245m TTAKLDEEKQEKFKKNIEGATKFLLSKLSDL---QFFVGESM-HDDGSLV 143

L.usitatissimum_Lus10033959_ LTAKLDDEQKEKFKKNIEAATKYLLSKLSDL---QFFVGESM-KDDATLV 143

P.trichocarpa_Potri.010G013400 LSEKLDEDQKEHFRKNIEGATKFLLSKIKDL---QFFVGESM-HDDGCLV 143

P.trichocarpa_Potri.008G226500 LSEKLDDENKEHFKKNIEGATKFLLSKIKDF---QFFVGESM-HDDSALV 143

G.max_Glyma09g04950_ LTPKLDAEQQELFKKHIEGATKYLLSKIKDF---QFFVGESM-GDDACLV 143

P.vulgaris_Phvul.009G248700_ LTAKLEGEQLESFKKHIEGATKYLLPKLKDS---QFFVGESM-HDDGCLV 142

G.raimondii_Gorai.005G060700_ LTPKLDAEKQESFKKNIEGATKFLLSKLKDL---QFFVGESM-HDDGCLV 143

S.bicolor_XP_002453140_ LTGKLEPEKADEFKKGIEGATKFLLSKLKDL---QFFVGESM-HDDGSLV 142

P.virgatum_Pavirv00039226m_ LTAKLDPEKADEFKKGIEGATKYLLSKLKDL---QFFVGESM-HDDGSLV 142

S.italica_Si026772m_ LTAKLEPEQQAEFKKGIEGATKYLLGKLKDL---QFFVGESM-HDDGSLV 143

O.sativa_Os11g43900_ LSAKLDAEKQEEFKKNIEGATKYLLGKLKDL---QFFVGESM-HDDGGLV 143

M.domestica_MDP0000164046_ LTPKLEGEGQEVFKKNIEAATKFLXGKLSDL---QFFVGESM-HDDGGLV 143

P.persica_ppa009639m_ LTPKLEGEKQEEFKKNIEGATKFLLSKLSDL---QFFVGESM-HDDGGLV 143

F.vesca_mrna06814.1-v1.0-hybri LTPKLEGEQQEIFKKNIEGATKFLLSKLSDL---QFFVGESM-GDDTSLV 143

V.vinifera_GSVIVT01017723001_ LTPKLEGEKQEEFKKNIEGATKFLLSKLSDL---QFFVGESM-HDDGSLV 142

R.communis_29726.m004052_ VTPKLDQETQEHFKKNIEGATKFLLSKLSDL---QFFVGESM-HDDGSLV 143

S.tuberosum_PGSC0003DMT4000635 LTPKLEGEAQEAFKKNIESATKFLLSKLKDF---QFFVGEGM-HDDSALV 143

S.lycopersicum_Solyc01g099780_ LTPKLEGETQEAFKKNIESATKFLLQKIKDL---QFFVGESM-HDDSALV 143

A.coerulea_Aquca_035_00202_ LASKLEGE--EVFKKNIEGAIKFLLSKIRDL---QFFVGVSM-NDDSSLV 140

A.coerulea_Aquca_003_00740_ LAPKLEGERQEVFKKNIEGATKFLLSKIKDL---QFFVGESM-SDDSSLV 142

C.sativus_Cucsa.253020_ LTPLLKGEQQEAFKSKIEGAVKFLLPKVKDL---QFFVGESM-ADDSAMV 143

C.melo_MELO3c006670p1__uniprot LTPLLQGEQQEAFKNKIEGAVKYLLPKVKDL---QFFVGESM-ADDSAMV 143

C.maxima_ABC02401_ LTPKLEGEKQEAFKKNIEGATKFLLPKLKDF---RFFVGESM-HDDSCIV 143

C.lunata_Cla021747 LTPKLEGEKQEAFKKNIEGATKYLLAKLKDL---QFFVGESM-ADDCSIV 143

A.thaliana_At3g16640_AAM66134_ LTPKLSEEDQAVFKKGIEGATKFLLPRLSDF---QFFVGEGM-HDDSTLV 143

B.rapa_Bra022172_ LTPKLNEEDQAAFKKGIEGATKFLLPKLNDF---QFFVGEGM-HDDSTLV 143

T.halophila_BAJ33998_ LTPKLDEEQQTAFKKGIEGATKFLLPKLGDF---QFFVGEGM-HDDSSLV 143

B.rapa_Bra001637_ LTPKLTPEQQEEFKKGIEGATKYLLPKLKDF---QFFVGEGM-HDDSTIV 143

A.lyrata_XP_002885160_ LTPKLNEEQQESFKKGIEGATKYLLPKLKDL---QFFVGESM-HDDSSLV 143

P.trichocarpa_Potri.005G024800 LTPKLEPEQQEVFKKNIEGATKFLFPKLKDF---QFFVGESM-HDDCSLV 142

A.coerulea_Aquca_017_00176_ LTPKLEGEAQEQFKKGIEGATKFLLPKLKDF---QFFVGESM-HDDSGLV 143

B.distachyon_Bradi4g10920_ LTPKLEADEQEVFKKNVEGATKFLLSKIKDL---QFFVGESM-GDDASVV 143

M.guttatus_Migut.N02086_ LIPKLESEKQDLFKKRIEGATKYLLSKIKDL---QFFVGESM-DDEGTVV 143

M.guttatus_Migut.G00151_ LSAKLEGEQQDQFKKTIEGATKYLLGKIKDL---QFFVGESM-HDDSTLV 143

P.patens_XP_001758666_ LTDLVPAERQASFKKDVEAAVKFLLSKLSDL---QFFVGESM-KDDSTIV 145

P.patens_XP_001757363_ LTPLVPAERQASFKKDVESAVKFLLSKLSDL---QFFVGESM-KDDSTMV 145

S.moellendorffi_179722_ LTEILPEEEKAEFKKNVEAAVKWILSKLNDF---QFFVGESM-KDDATYV 143

Z.mays_GRMZM2G108474_T01_ LTAVLEPEKADEFKKGVEGATKFLLSKLKDL---QFFVGESM-KDDASVV 142

C.subellipsoidea_C-169_65285_ VLEKLPADQQEDFKAKSQPAIKMLMGKIKEL---QFFLGESM-DQEGTLI 141

C.merolae_CMQ113C_ LKEYLENENPSRVAAFMEGMKEWVPKMLKEFDEYEFYMGPSC-DPDALLV 144

: . : : : : .*::* . : :

C.sativ_Cucsa.181820_ FAYYKEGATDPTFIYIAYGLKEVKC 168

C.melo_MELO3C015297P1__uniprot FAYYKEGATDPTFIYIAYGLKEVKC 168

C.lunata_Cla005200 FAYYKEGATDPTFIYIAYGLKEVKC 168

G.max_Glyma10g29240_ FAYYKEGATDPTFIYFAYGLKEIKC 168

R.communis_30128.m008835_ FAYYKEGATDPTFLYFAYGLKEVKC 168

M.esculenta_cassava4.1_017738m FAYYKEGSADPTFLYFAYGLKEVKC 168

V.vinifera_GSVIVT01031135001_ FAYYKEGATDPTFLYFAHGLKEIKC 168

M.truncatula_Medtr1g083350_ FAYYKDGATDPTFLYFAYGLKEIKC 168

P.vulgaris_Phvul.007G197200_ FAYYKEGATNPTFIYFAYGLKEIKC 168

M.truncatula_Medtr6g071090_ FAYYKEGATNPTFLYFA-------- 159

M.esculenta_cassava4.1_017756m FAYYKEGSTEPTFLYFAYGLKEIKC 168

A.thaliana_At3g05540_AAF26143_ FAYYREGATDPTFLYLAYGLKEIKC 168

A.lyrata_XP_002884515_ FAYYREGAADPTFLYLAYGLKEIKC 156

T.halophila_10022380m_ FAYYKEGATDPTFLYLAYGLKEIKC 168

G.raimondii_Gorai.013G26000_ FAYYKDGATNPTFLYFPYGLKEVKC 168

G.raimondii_Gorai.007G300300_ FAYYKDGATDPTFLYFAYGLKEIKC 168

C.clementina_Ciclev10002699m_ LAYYKEGATDPTFLYFAYGLKEVKC 168

C.sinensis_1.1g030941m_ FAYYKEGATDPTFLYIADALKEVKC 168

C.clementina_Ciclev10006071m_ FAYYKEGATDPTFLYMADALKEVKC 168

M.esculenta_cassava4.1_025245m FAYYKEGATDPTFLYFAYALKEVKC 168

L.usitatissimum_Lus10033959_ FAYYKDGAADPTFLYLPQALKEVKC 168

P.trichocarpa_Potri.010G013400 FAYYKEGATDPTFLYFAPSLKEVKC 168

P.trichocarpa_Potri.008G226500 LAYYKEGATDPTFLYFGHALKEVKC 168

G.max_Glyma09g04950_ FAYYKDGAADPTFLYFAYALKEVKC 168

P.vulgaris_Phvul.009G248700_ LAYYKDGATDPTFLYFAYALKEVKC 167

G.raimondii_Gorai.005G060700_ FAYYKDGAVDPTFLYFAYALKEVKC 168

S.bicolor_XP_002453140_ FAYYKEGATDPTFLYFAHGLKEIKC 167

P.virgatum_Pavirv00039226m_ FAYYKDGATDPTFLYFAHGLKEIKC 167

S.italica_Si026772m_ FAYYKDGATDPTFLYFAHGLKEIKC 168

O.sativa_Os11g43900_ FAYYKDGATDPTFLYFSHGLKEVKC 168

M.domestica_MDP0000164046_ FAYYKEGATDPTFIYFGHGLKEVKC 168

P.persica_ppa009639m_ FAYYKEGATDPTFIYFAHGLREVKC 168

F.vesca_mrna06814.1-v1.0-hybri FAYYKEGATDPTFIYFAHGLKEVKC 168

V.vinifera_GSVIVT01017723001_ FAYYKDGATDPTFLYFGHGLKEIKC 167

R.communis_29726.m004052_ FAYYKDGAADPTFLYFAHGLKEIKC 168

S.tuberosum_PGSC0003DMT4000635 FAYYKDGSADPTFLYLAPGLKEIKC 168

S.lycopersicum_Solyc01g099780_ FAYYKDGSADPTFLYLAPGLKEIKC 168

A.coerulea_Aquca_035_00202_ FAYYKD----------AHGLKEIKC 155

A.coerulea_Aquca_003_00740_ FAYYKDGATDPTFLYFAHGLKEVKC 167

C.sativus_Cucsa.253020_ FAYYKEGATDPTFLYIAPGLKEVKC 168

C.melo_MELO3c006670p1__uniprot FAYYKEGATDPTFLYIAPGLKEVKC 168

C.maxima_ABC02401_ FAYYREGATDPTFLYLAPALKEVKC 168

C.lunata_Cla021747 FAYYKEGATEPTFLYLAPGLKEVKC 168

A.thaliana_At3g16640_AAM66134_ FAYYKEGSTNPTFLYFAHGLKEVKC 168

B.rapa_Bra022172_ FAYYKEGATNPTFLYFGHGLKEVKC 168

T.halophila_BAJ33998_ FAYYKEGATNPTFLYFAHGLKEVKC 168

B.rapa_Bra001637_ FAYYKEGATNPTFLYFAHGLKEVKC 168

A.lyrata_XP_002885160_ FAYYKDGATNPTFLYFAHGLKEVKC 168

P.trichocarpa_Potri.005G024800 LAYYKEGATDPTFLYFAHGLKEVKC 167

A.coerulea_Aquca_017_00176_ LAYYKEGATDPTFLYFAHGLKEVKC 168

B.distachyon_Bradi4g10920_ FAYYKEGATDPTFLYFAHGLKEVKC 168

M.guttatus_Migut.N02086_ FAYYKESATDPTFLYLAYGLKEVKC 168

M.guttatus_Migut.G00151_ FAYYKDGAADPTFLYFAHGLKEIKC 168

P.patens_XP_001758666_ FAYYKDGAANPTFLYFGDALKEVKC 170

P.patens_XP_001757363_ FAYYKEGQSNPTFLYFRDALKEVKC 170

S.moellendorffi_179722_ LAYYKEGRSDPTFIYFKHALKEVKC 168

Z.mays_GRMZM2G108474_T01_ FAYYKDGATNPTFLYFSHGLKEIKC 167

C.subellipsoidea_C-169_65285_ FAYYADGAAEPKFLYPKYALQEIKA 166

C.merolae_CMQ113C_ LAKYEGESHYPTFLYLKDGLAEEKF 169
