# Supplementary material for: Structural divergence of plant TCTPs
Source: Front Plant Sci. 2014 Jul 29;5:361. doi: 10.3389/fpls.2014.00361 (PMC4114181; doi:10.3389/fpls.2014.00361)
Supplement: Figure S2 — Phylogenetic analysis of the TCTP in plants. C. maxima translationally controlled tumor protein (TCTP) sequence (GenBank Accession No. ABC02401) was retrieved form NCBI (http://www.ncbi.nlm.nih.gov) and used as query. Protein homologs were identified by BLAST search against the phytozome database (http://phytozome.net). Full-length protein sequences were aligned with MUSCLE (http://www.ebi.ac.uk/Tools/msa/muscle). ProtTest program (PROTTEST 2.4, Abascal et al., 2005) was used for the selection of the model of protein evolution that best fitted the set of sequences. Evolutionary analysis was conducted in PhyML 3.0 (Guindon et al., 2010) and MEGA5. The evolutionary phylogenetic reconstruction (shown as dendrogram) was inferred using the LG+G evolution method and the following parameters: number of substitution rate categories 4, gamma shape parameter 0.642. Bootstrap values higher than 60% are shown (1000 replicates). The analysis involved 55 amino acid sequences. [file DataSheet2.DOC]

Figure S2

Gutiérrez-Galeano *et al*., 2014
