# Supplementary material for: Structural divergence of plant TCTPs
Source: Front Plant Sci. 2014 Jul 29;5:361. doi: 10.3389/fpls.2014.00361 (PMC4114181; doi:10.3389/fpls.2014.00361)

Figure S3
Gutiérrez-Galeano *et al.*, 2014
AtTCTP1-like proteins

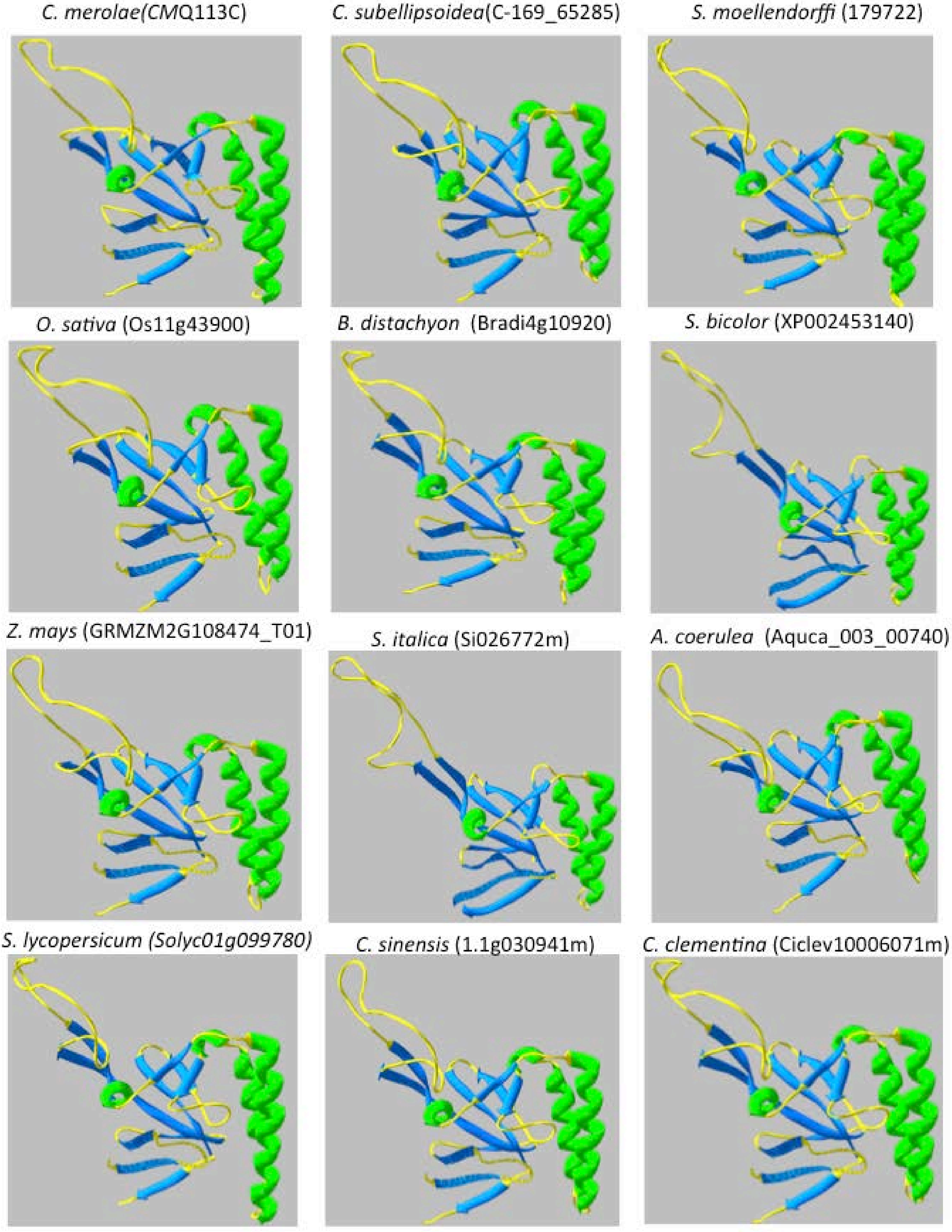

Figure S3
Gutiérrez-Galeano *et al.*, 2014
(Continued)
AtTCTP1-like proteins

G. raimondii (Gorai.005G060700)

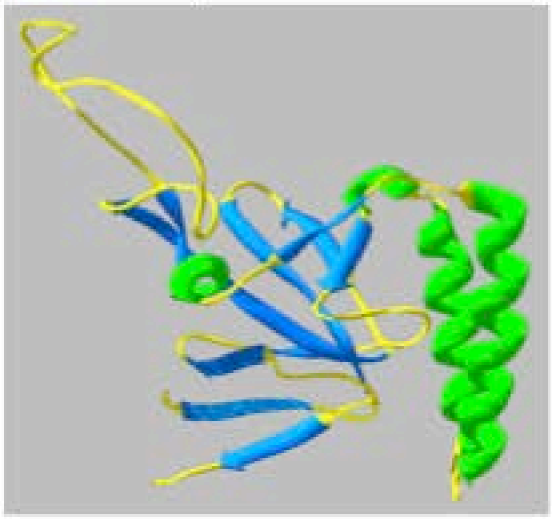

T. halophila (BAJ33998)

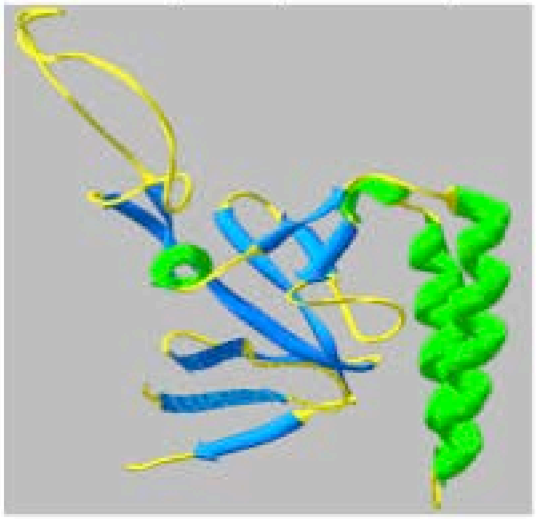

B. rapa (Bra022172)

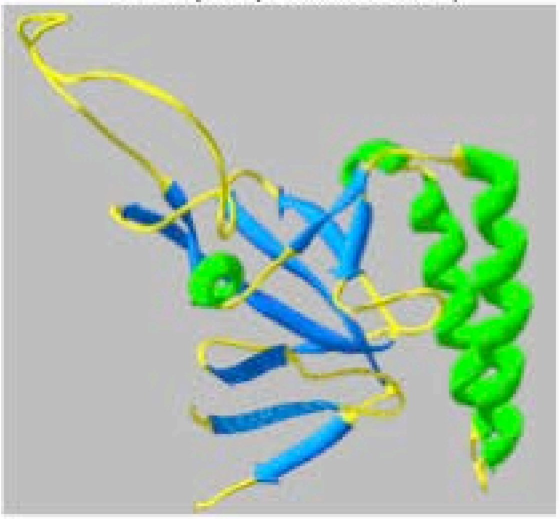

A. lyrata (XP_002885160)

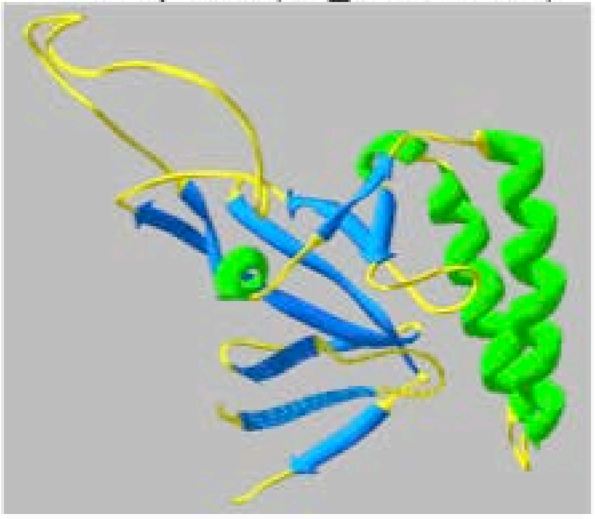

A. thaliana (At3g16640_AAM66134)

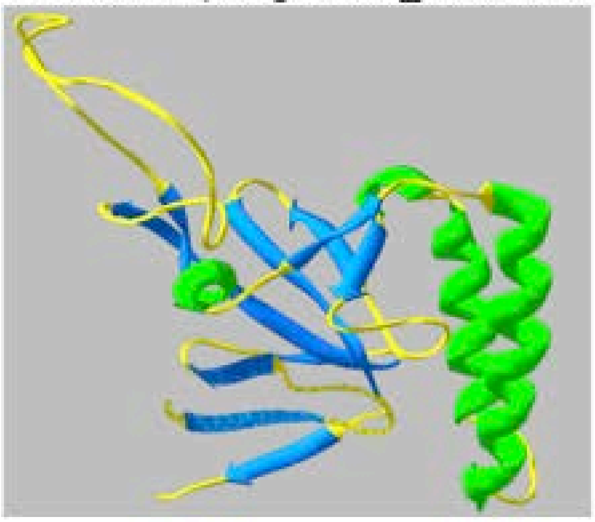

M. truncatula (Medtr6g07190)

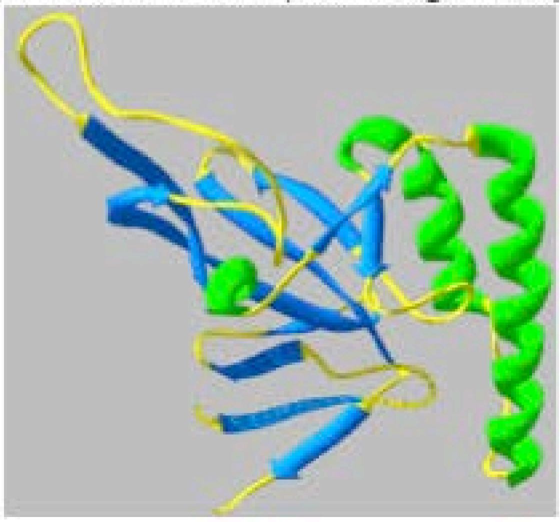

*F. vesca (*rna06814.1-v1.0-hybrid)

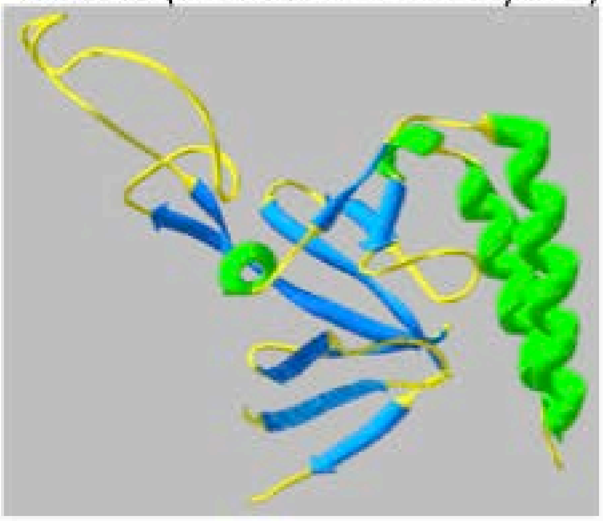

P. persica (ppa009639m)

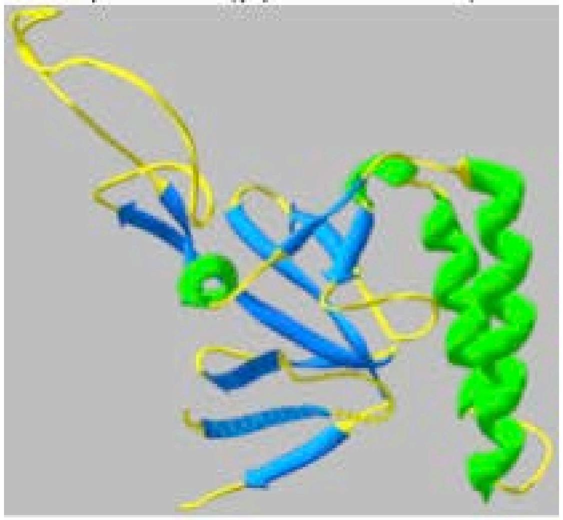

C. sativus (Cucsa.253020)

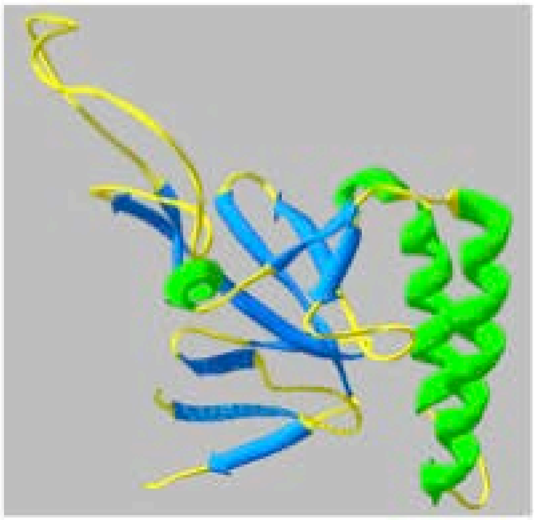

Figure S3
Gutiérrez-Galeano *et al.*, 2014
(Continued)
AtTCTP1-like proteins

C. melo (MELO3c006670p1)

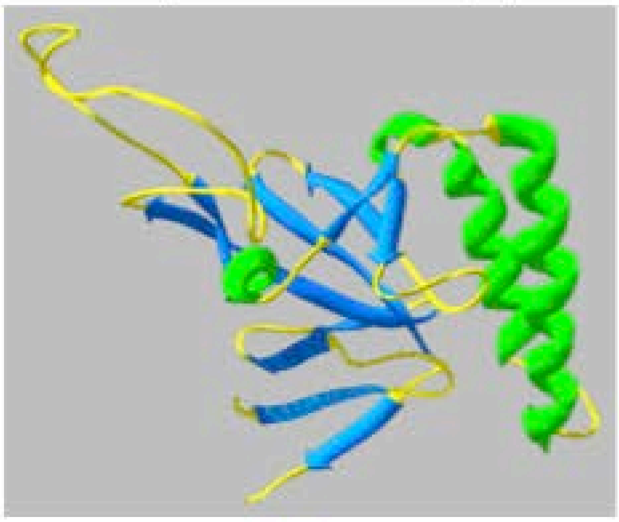

C. lanatus (Cla021747)

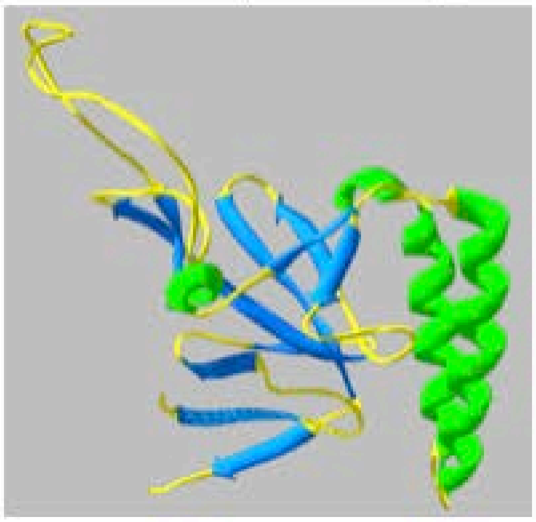

P. trichocarpa (Potri.008G226500)

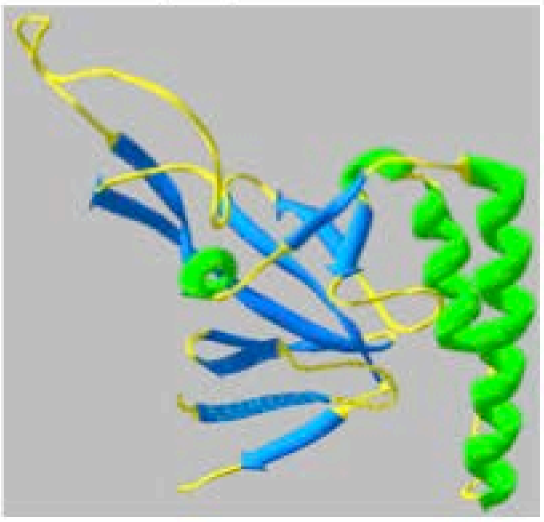

P. trichocarpa (Potri.005G024800)

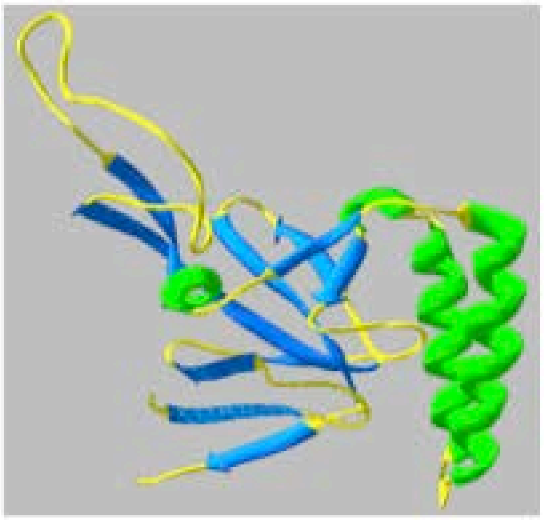

L. usitatissimum (Lus10033959)

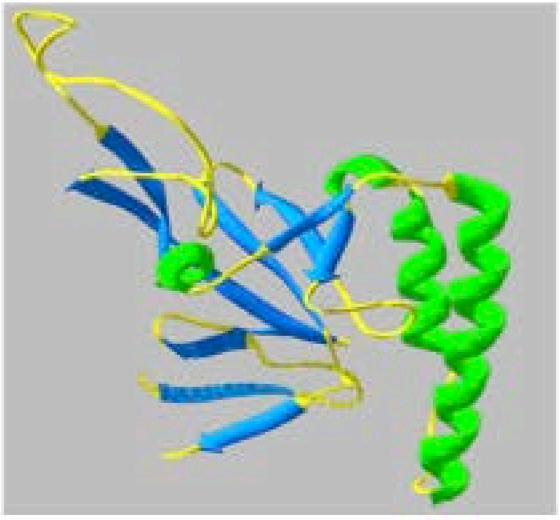

M. esculenta (cassava4.1_025245m)

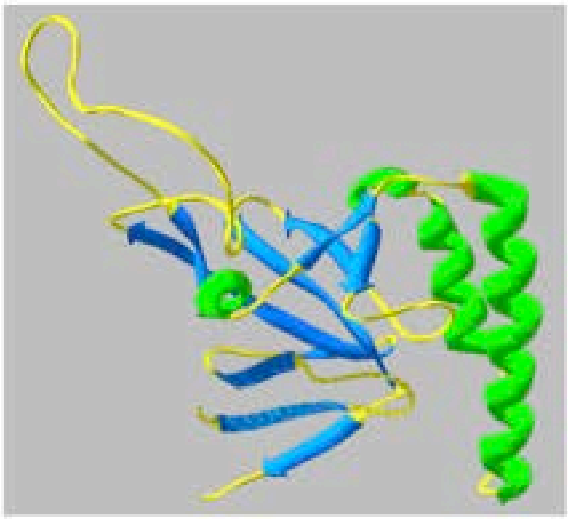

B. rapa (Bra022172)

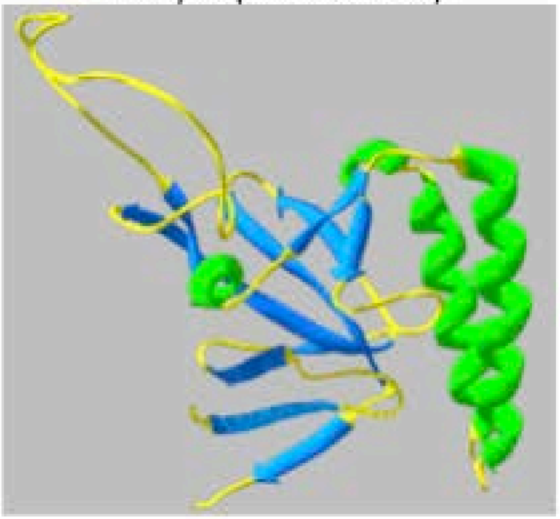

C. rubella EOA33129.1

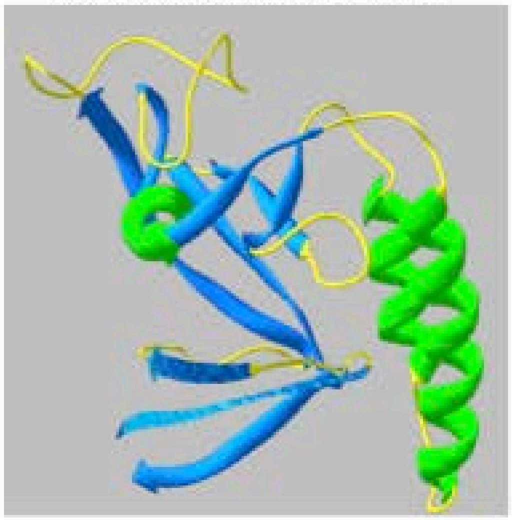

Figure S4
Gutiérrez-Galeano *et al.*, 2014
CmTCTP-like proteins

P. patens (XP_001758666)

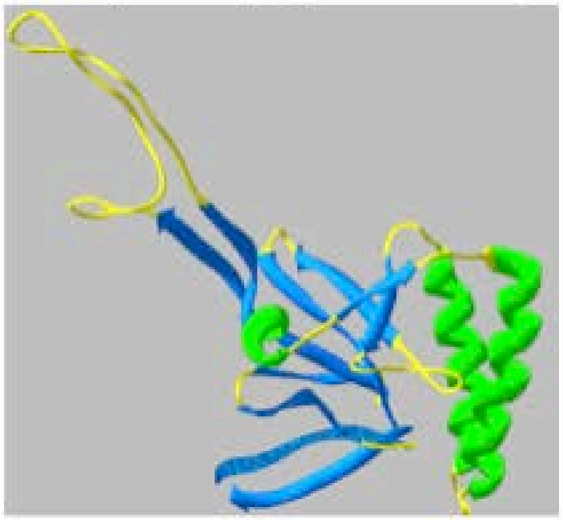

A. coerulea (Aquca_035_00202)

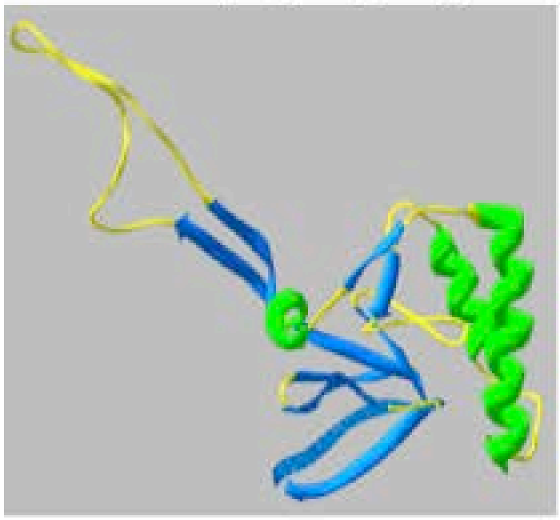

A. coerulea (Aquca_017_00176)

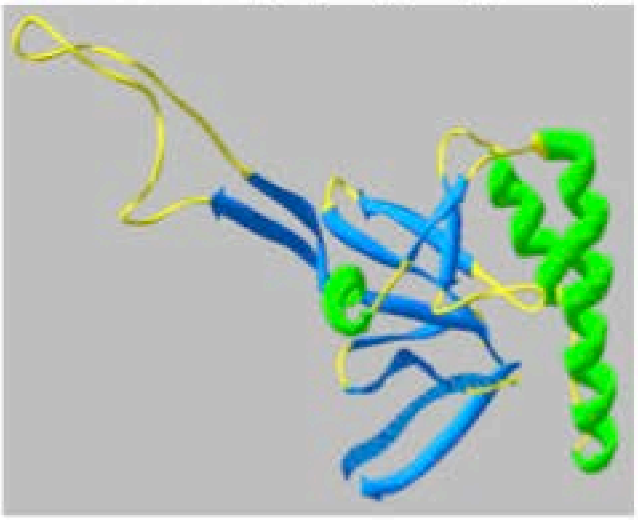

P. virgatum(Pavirv00039226m)

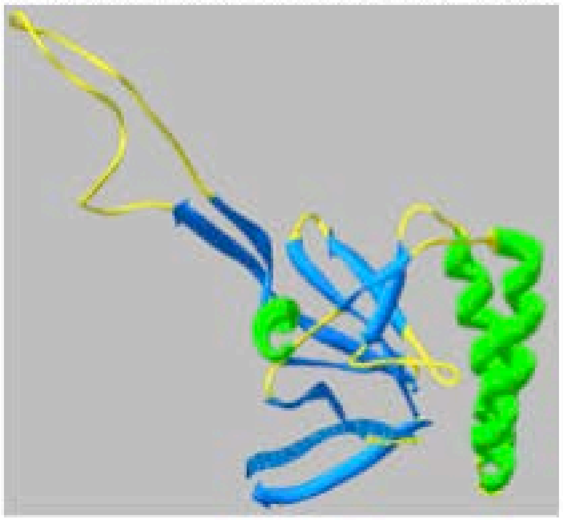

S. tuberosum (PGSC0003DMT400063579)

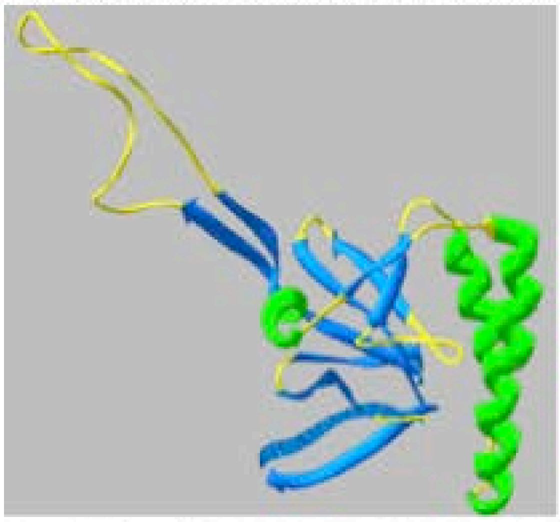

M. guttatus (Migut.N02086)

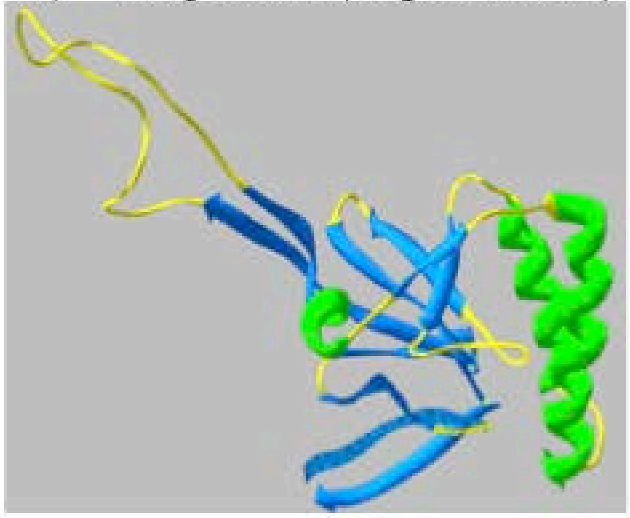

M. guttatus (Migut.G00151)

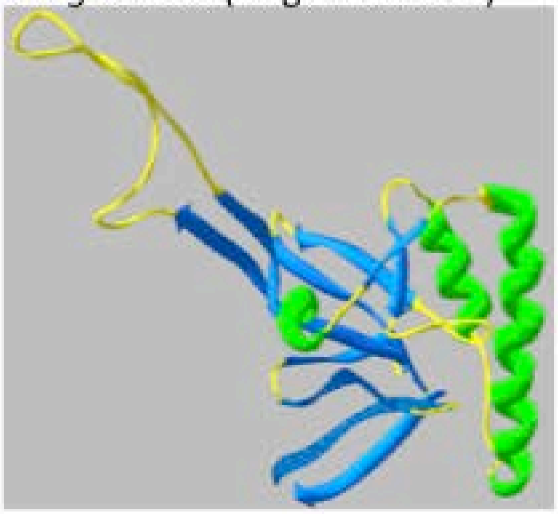

V. vinifera (GSVIVT01017723001)

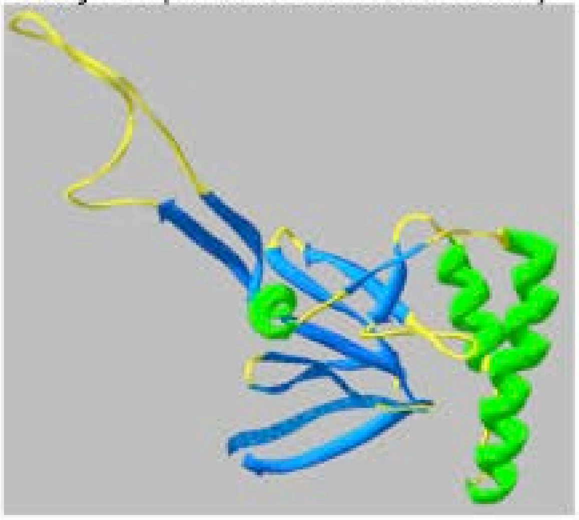

V. vinifera (GSVIVT01031135001)

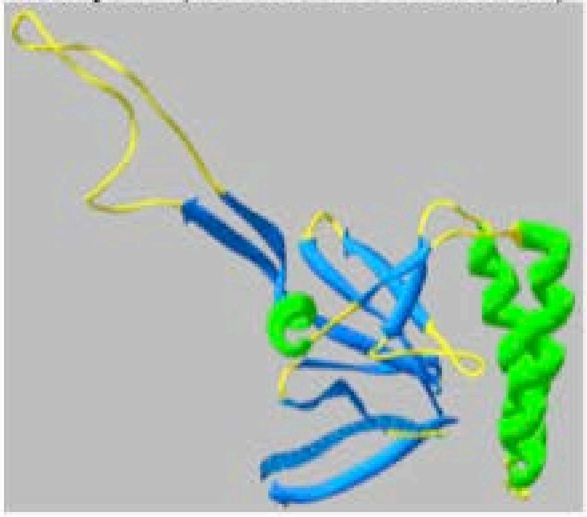

C. clementina (Ciclev10006071m)

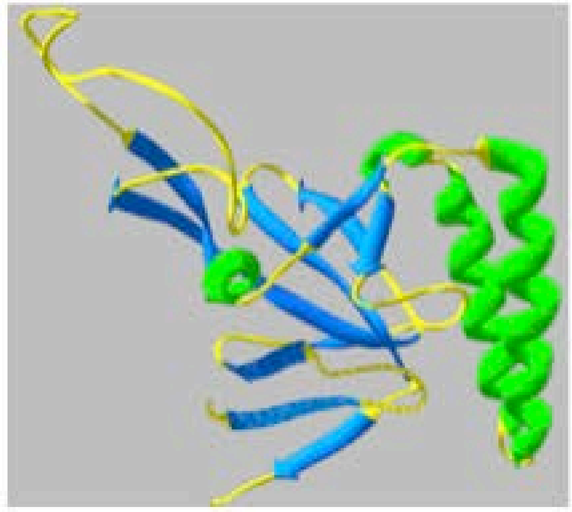

G. raimondii (Gorai.013G26000)

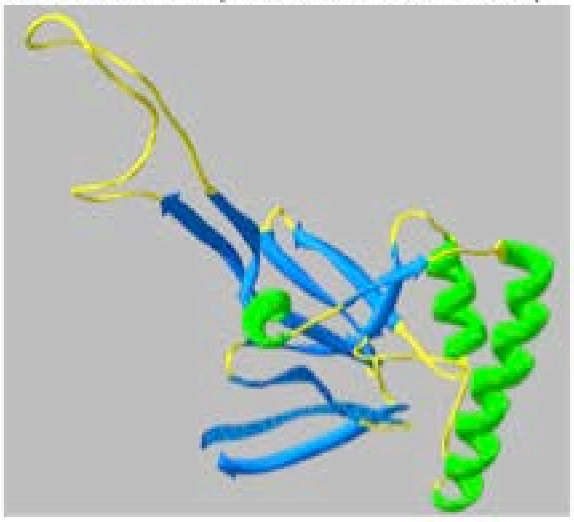

G. raimondii (Gorai.007G300300)

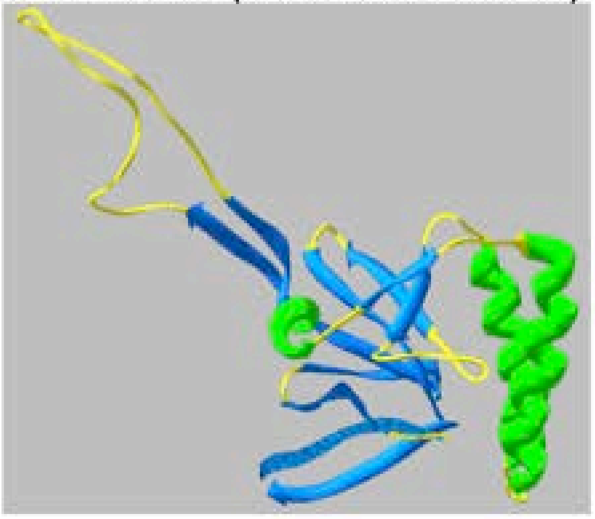

Figure S4

Gutiérrez-Galeano *et al.*, 2014

Continued

CmTCTP-like proteins

T. halophila (10022380m)

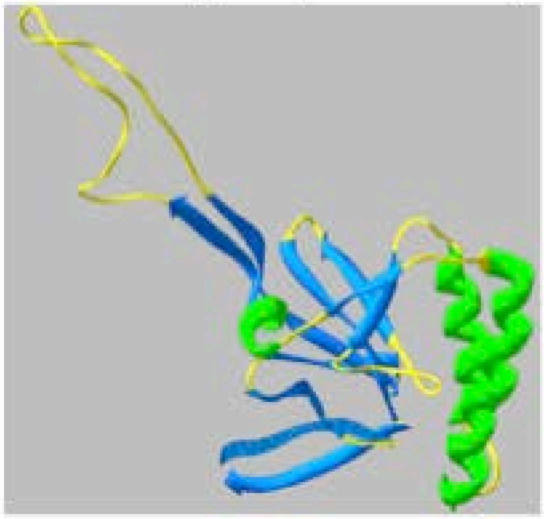

B. rapa (Bra001637)

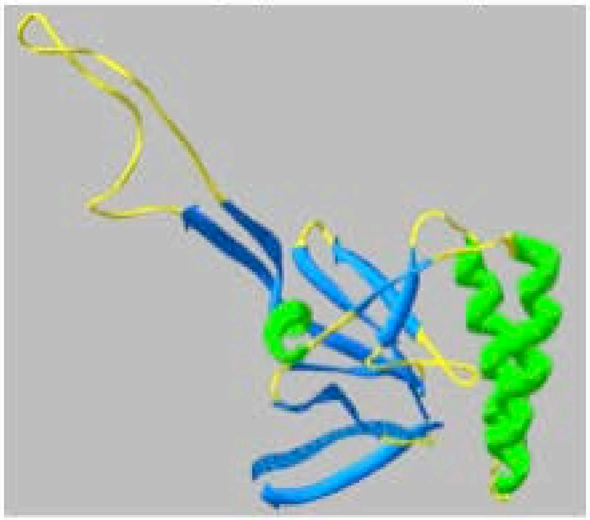

A. lyrata (XP_002884515)

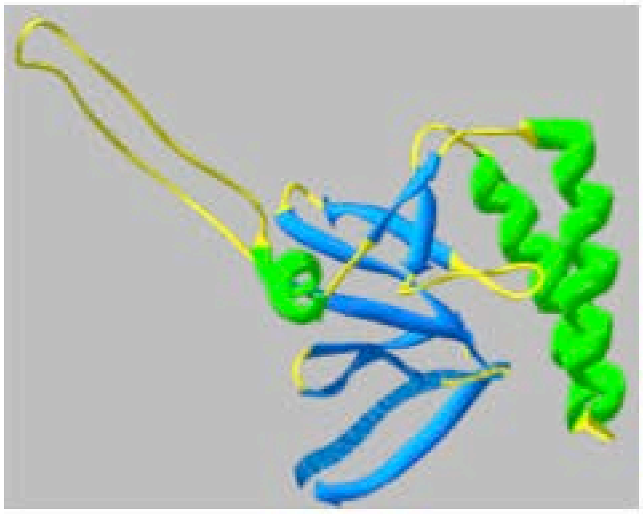

A. thaliana (At3g05540_AAF26143)

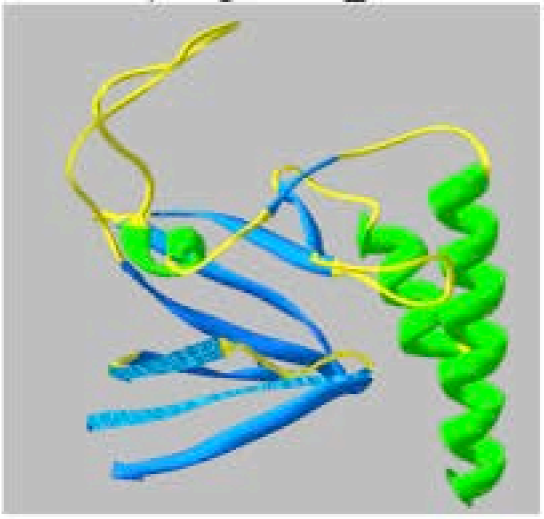

G. max (Glyma09g04950)

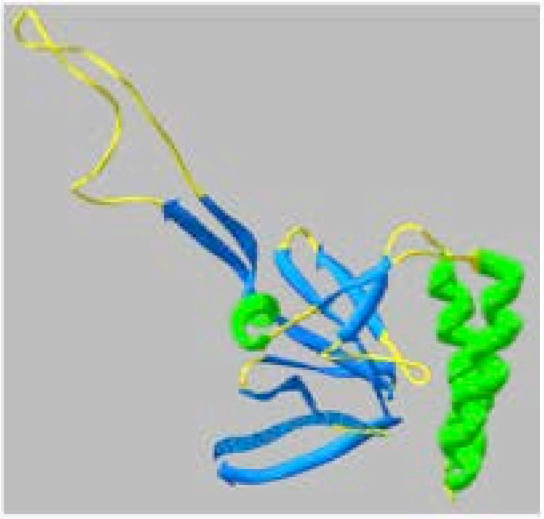

G. max (Glyma10g29240)

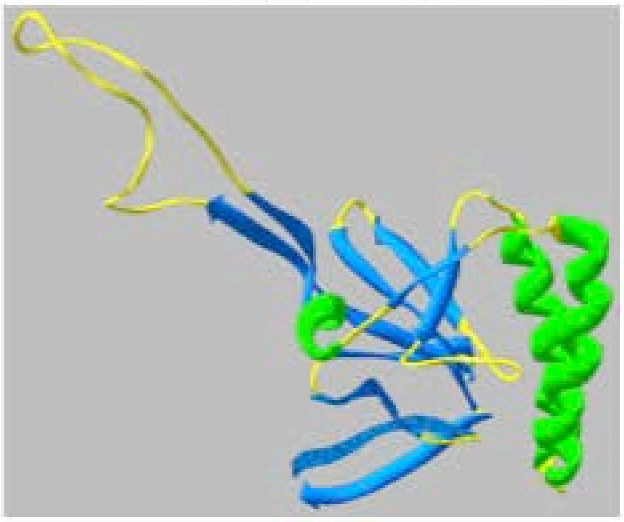

P. vulgaris (Phvul.009G248700)

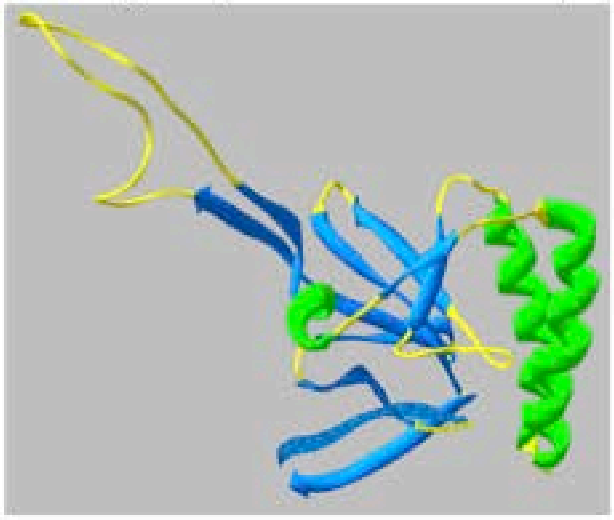

P. vulgaris (Phvul.007G197200)

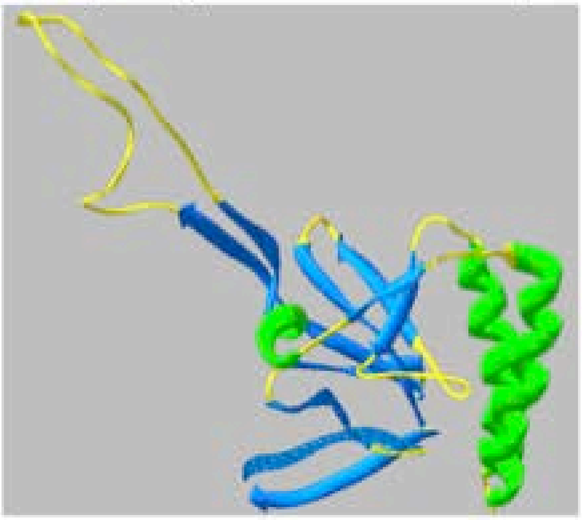

M. truncatula (Medtr6g07190)

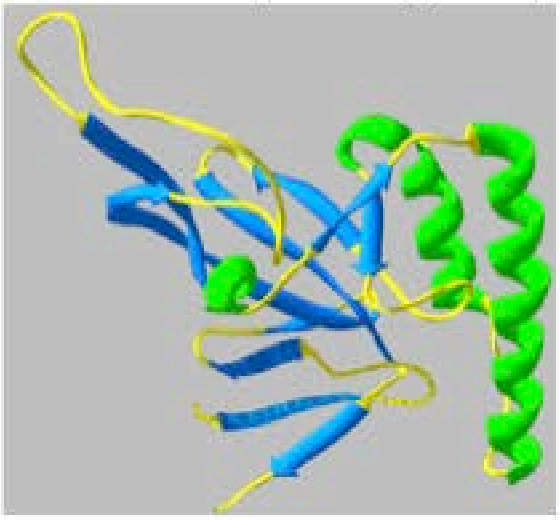

Figure S4

Gutiérrez-Galeano *et al.*, 2014

Continued

CmTCTP-like proteins

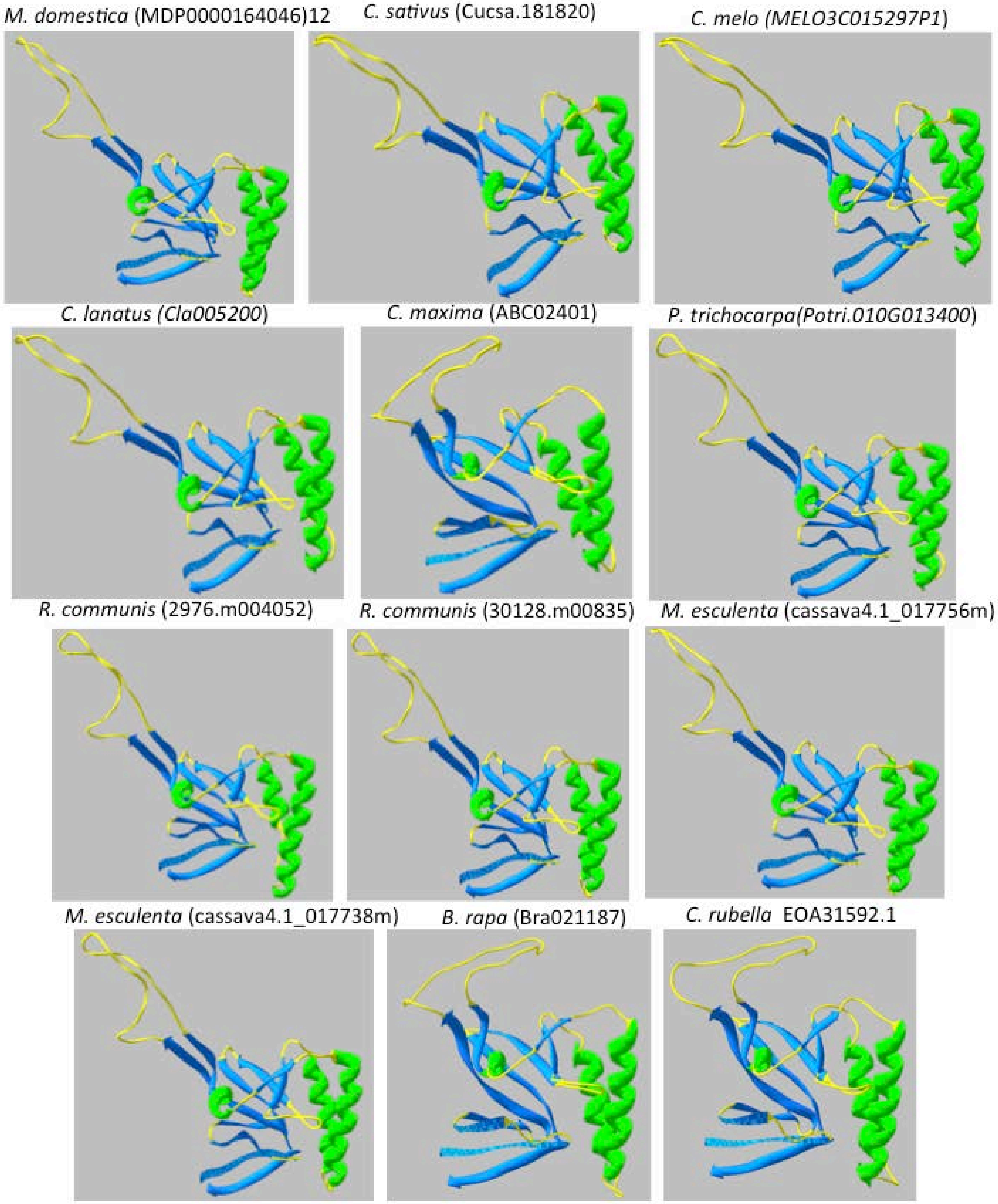

Figure S4
Gutiérrez-Galeano *et al.*, 2014
Continued
CmTCTP-like proteins

C. papaya
evm.model.supercontig_1597.1

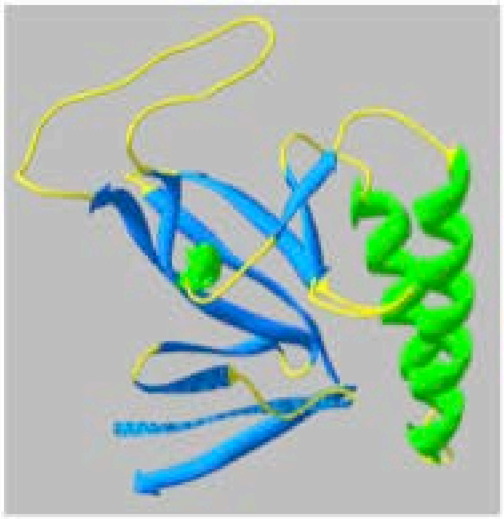

C. papaya
evm.model.supercontig_327.3

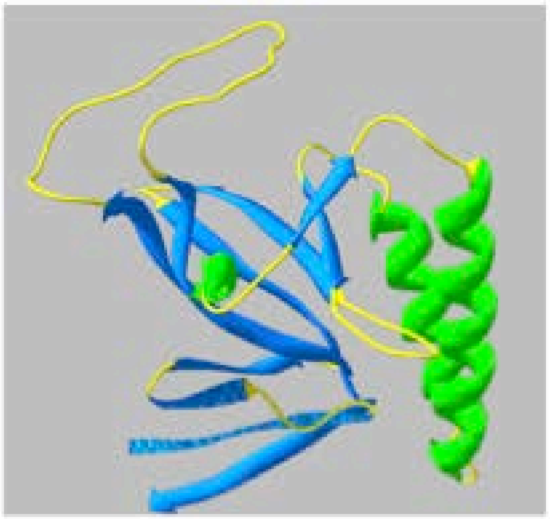

P. patens (XP_001757363.1)

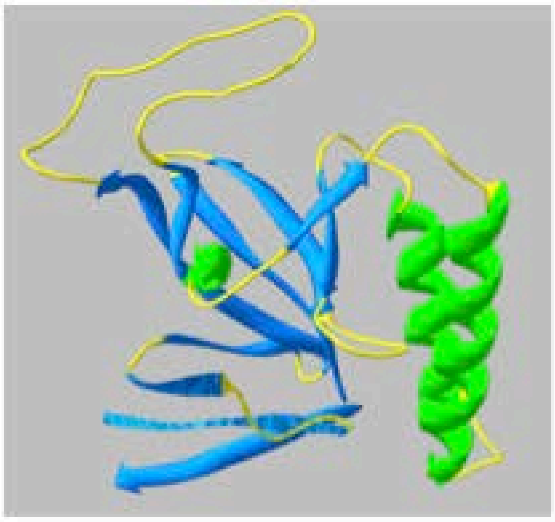

Supplement: Figure S3 — Predictive 3D structure comparison of AtTCTP-like proteins. Full-length protein sequences were selected from phytozome database (http://phytozome.net) and refined through a comparison with NCBI database (http://www.ncbi.nlm.nih.gov). Amino acid sequences were submitted to the automated protein structure homology-modeling program server SWISS-MODEL (http://swissmodel.expasy.org/), this server builds a model for each protein target using as templates homologous protein structures which have been experimentally proved (Arnold et al., 2006; Kiefer et al., 2009). Swiss PDB Viewer application (http://www.expasy.org/spdbv/) was used for visualizing predictive 3D structures (Guex and Peitsch, 1997). [file DataSheet3.PDF]
