# Supplementary material for: Structural divergence of plant TCTPs
Source: Front Plant Sci. 2014 Jul 29;5:361. doi: 10.3389/fpls.2014.00361 (PMC4114181; doi:10.3389/fpls.2014.00361)
Supplement: Supplementary file 4 [file Table1.PDF]

Additional Table. Percentage of regeneration induced by tobacco transformation with CmTCTP overexpression construct compared to AtTCTP1 and empty vector (control)

Construct	% of plant regeneration<br>[plants regenerated±SD / leaf explants]
Control (empty vector)	1% [1±1/85]*
35S::CmTCTP -GFP	52%[42±4/80]
35S::AtTCTP1-GFP	3%± [3±3/85]*

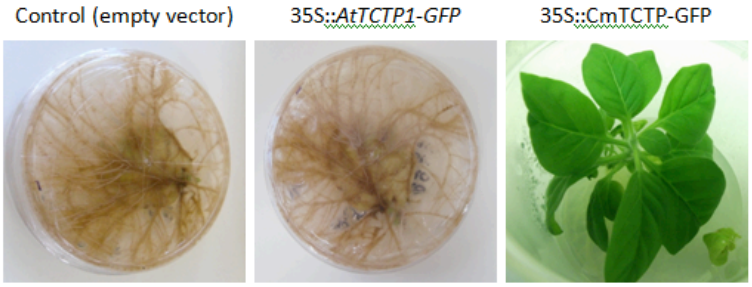

Authors:

Toscano-Morales, R., Xoconostle-Cázares, B., Cabrera-Ponce, J.L., Hinojosa-Moya, J.J., Guevara-González, R.G., Ruiz-Medrano, R. 2014. Submitted
